# Amoeba Genome Reveals Dominant Host Contribution to Plastid Endosymbiosis

**DOI:** 10.1093/molbev/msaa206

**Published:** 2020-08-13

**Authors:** Duckhyun Lhee, JunMo Lee, Khaoula Ettahi, Chung Hyun Cho, Ji-San Ha, Ya-Fan Chan, Udi Zelzion, Timothy G Stephens, Dana C Price, Arwa Gabr, Eva C M Nowack, Debashish Bhattacharya, Hwan Su Yoon

**Affiliations:** 1 Department of Biological Sciences, Sungkyunkwan University, Suwon, Korea; 2 Department of Oceanography, Kyungpook National University, Daegu, Korea; 3 Department of Biochemistry and Microbiology, Rutgers University, New Brunswick, NJ; 4 Department of Entomology, Center for Vector Biology, Rutgers University, New Brunswick, NJ; 5 Microbiology and Molecular Genetics Graduate Program, Rutgers University, New Brunswick, NJ; 6 Institut für Mikrobielle Zellbiologie, Heinrich-Heine-Universität, Düsseldorf, Germany

**Keywords:** chromatophore, gene coexpression analysis, *Paulinella*, photosynthetic amoeba, primary endosymbiosis

## Abstract

Eukaryotic photosynthetic organelles, plastids, are the powerhouses of many aquatic and terrestrial ecosystems. The canonical plastid in algae and plants originated >1 Ga and therefore offers limited insights into the initial stages of organelle evolution. To address this issue, we focus here on the photosynthetic amoeba *Paulinella micropora* strain KR01 (hereafter, KR01) that underwent a more recent (∼124 Ma) primary endosymbiosis, resulting in a photosynthetic organelle termed the chromatophore. Analysis of genomic and transcriptomic data resulted in a high-quality draft assembly of size 707 Mb and 32,361 predicted gene models. A total of 291 chromatophore-targeted proteins were predicted in silico, 208 of which comprise the ancestral organelle proteome in photosynthetic *Paulinella* species with functions, among others, in nucleotide metabolism and oxidative stress response. Gene coexpression analysis identified networks containing known high light stress response genes as well as a variety of genes of unknown function (“dark” genes). We characterized diurnally rhythmic genes in this species and found that over 49% are dark. It was recently hypothesized that large double-stranded DNA viruses may have driven gene transfer to the nucleus in *Paulinella* and facilitated endosymbiosis. Our analyses do not support this idea, but rather suggest that these viruses in the KR01 and closely related *P. micropora* MYN1 genomes resulted from a more recent invasion.

## Introduction

**Fig. 1.**
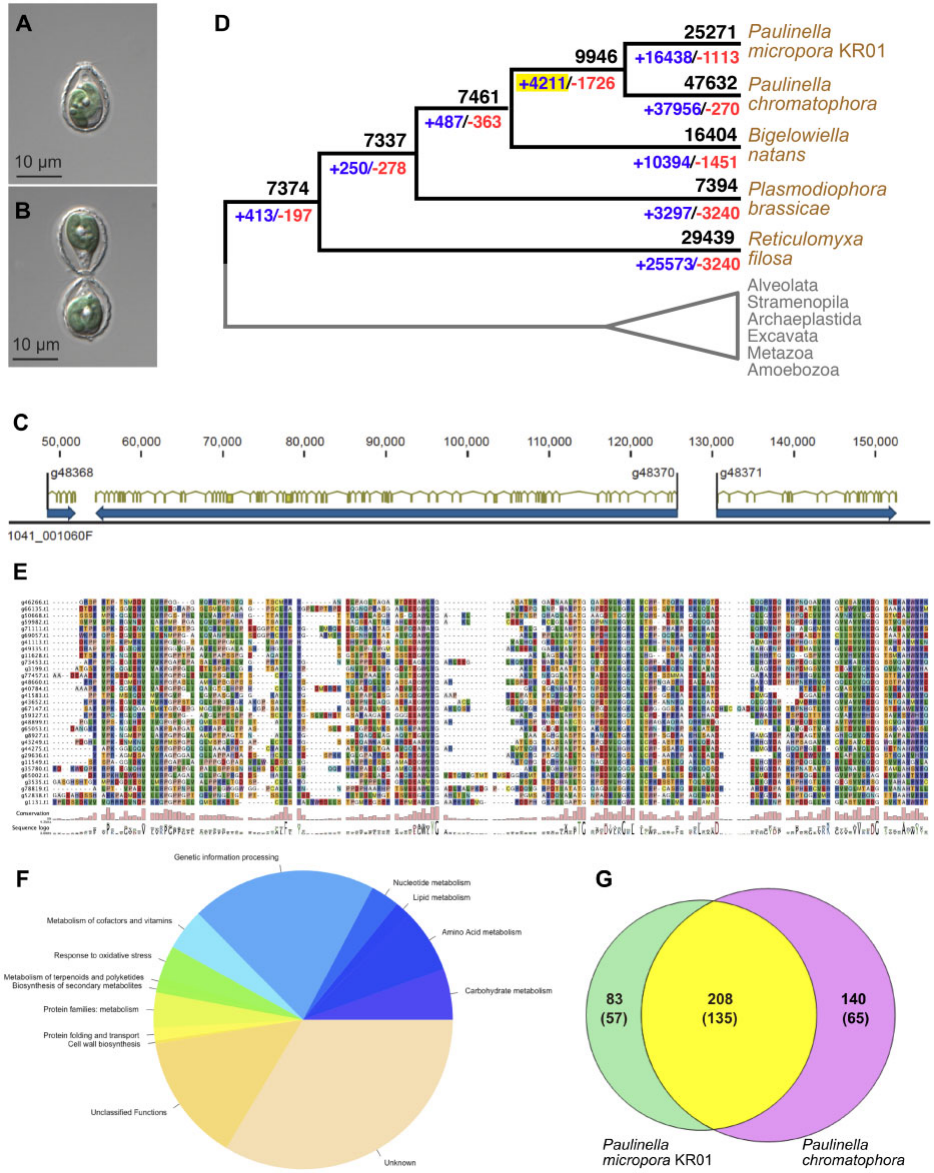
Genome analysis of the photosynthetic amoeba *Paulinella micropora* KR01. (*A*) Light micrograph of a mature KR01 cell showing the two sausage-shaped chromatophores. The cell is covered in a siliceous shell made of scales that are visible in cross-section. (*B*) A newly formed daughter cell connected to the mother cell. (*C*) Region of a typical contig (1041_001060F) from the KR01 genome showing the complex intron–exon (filled yellow boxes) structure of many genes (filled blue boxes) in this species. The position in base pairs is shown above the contig. (*D*) OGF evolution in rhizarian species. Ancestral OGF numbers (black) with gain (blue) and loss (red) are indicated at the branch. The yellow box indicates OGFs gained in the common ancestor of photosynthetic *Paulinella* species. (*E*) Alignment of KR01 crTPs from the long import candidates identified using BLAST hits to validated crTPs in *Paulinella chromatophora*. These crTPs were used to build and calibrate an HMM profile used to identify KR01 long import candidates. (*F*) Functional annotation of the predicted 291 import candidates generated using KAAS and categorized based on KEGG functional categories. (*G*) Comparison of the in silico predicted chromatophore import candidates in KR01 to predicted import candidates in *P. chromatophora* (proteins found enriched in the chromatophore proteome analysis as well as HMM in silico predicted chromatophore import candidates). The number of annotated proteins is shown in parentheses below the total number.

As sites of primary production, eukaryotic photosynthetic organelles, plastids, are the powerhouses of many aquatic and terrestrial ecosystems (Bhattacharya et al. 2004; Keeling 2010). Plastids originated via primary endosymbiosis >1 Ga in the ancestor of the Archaeplastida, whereby a cyanobacterium was captured and retained as the photosynthetic organelle (Yoon et al. 2004; Parfrey et al. 2011). The plastid in red and green algae was subsequently engulfed by other eukaryotes via secondary or tertiary endosymbiosis (Bhattacharya et al. 2004). This process gave rise to a vast array of extant photosynthetic lineages such as diatoms, dinoflagellates, chlorarachniophytes, and euglenids. Despite its great impact on eukaryote evolution and diversity, the understanding of primary endosymbiosis, particularly its early stages, remains limited due to the long timespan since this event took place. For this reason, the independent and more recent primary endosymbiosis in the rhizarian amoeba, *Paulinella* (fig. 1*A* and *B*), provides a unique opportunity for studying plastid evolution. In contrast to the highly reduced plastid genomes of algae and plants (i.e., 100–200 kb in size in Archaeplastida), the α-cyanobacterium-derived photosynthetic compartment (referred to as the chromatophore [plastid]) in phototrophic *Paulinella* is in a less derived stage of organelle evolution and is ∼1 Mb in size (Nowack et al. 2008; Reyes-Prieto et al. 2010). The α-cyanobacteria contain the diverged *Prochlorococcus* clade with some (free-living) species having undergone extensive genome reduction (e.g., 1.8 Mb genome size, 1,884 protein-coding regions in *Prochlorococcus marinus* SS120; Dufresne et al. 2003). However, the chromatophore is resolved in phylogenetic trees as a sister to the entire *Synechococcus*/*Prochlorococcus* clade or in a basal position within *Synechococcus* (Yoon et al. 2006; Marin et al. 2005). Calculation of the number of orthologous gene families (OGFs) in the chromatophore ancestor suggests this number is 2,502 (Lhee et al. 2019). Therefore, it is important to note that although the likely chromatophore donor had a smaller genome than many cyanobacteria, it was not a nearly minimal genome as reported for the low-light adapted *Prochlorococcus marinus* SS120 (Dufresne et al. 2003). In comparison, the core genome of marine *Synechococcus* comprises 1,572 gene families that include a wide diversity of ecotypes (Dufresne et al. 2008).

In photosynthetic *Paulinella* species, genes encoded by the previously free-living endosymbiont were lost outright, retained in the organelle genome, or transferred to the host nuclear genome via endosymbiotic gene transfer (EGT) (Martin and Herrmann 1998; Reyes-Prieto et al. 2006). In addition, horizontal gene transfer (HGT)-derived bacterial genes in the amoeba nucleus encode proteins that fill gaps in critical chromatophore pathways (Nowack et al. 2016). Proteins, both native and those derived via HGT, that are synthesized in the cytoplasm of the amoeba are transported into the chromatophore where they express their functions (Singer et al. 2017). Current knowledge about photosynthetic *Paulinella* species indicates a chimeric nuclear gene inventory that evolved to rescue chromatophore functions and enable the phototrophic lifestyle. To advance knowledge about primary EGT, HGT, host genome, and chromatophore evolution in photosynthetic *Paulinella*, we generated a draft genome assembly and transcriptome data from *Paulinella micropora* strain KR01 (hereafter KR01) (Lhee et al. 2017). Our analyses demonstrate the dynamic early stages of plastid integration and identify the massive contribution by putatively novel (i.e., using standard sequence comparison methods), so-called “dark” genes (Stephens et al. 2018; Cleves et al. 2020) to photosynthetic organelle establishment. Here, dark genes or proteins lack a MMseqs2 hit at the *e*-value cutoff ≤1e^−5^ against the nonredundant NCBI database and comprise de novo gene originations in the *Paulinella* clade (and potentially its sister species for which we have no genome data) or are genes too highly diverged to identify their homologs with known functions.

## Results and Discussion

### KR01 Genome Features

De novo assembly, after removing bacterial and organelle DNA, resulted in 77,048 KR01-derived contigs with N50 = 143,028 bp and a total length of 707 Mb (supplementary tables S1–S3, Supplementary Material online). In contrast, the nuclear genome in the sister lineage *Paulinella chromatophora* is ∼10 Gb in size and thus, has not yet been successfully assembled (Nowack et al. 2016). The KR01 genome GC-content is 44% and contains 76% repetitive sequences (supplementary table S4, Supplementary Material online) that were masked prior to protein-coding gene prediction using BRAKER2, resulting in a conservative estimate of 32,361 genes. A total of 89% of these predicted genes (28,871) contain multiple exons (e.g., fig. 1*C*; avg. = 10 exons/gene) and 3,490 were single exon predictions (table 1). The mean length of introns in KR01 (882 bp) is significantly greater than in other rhizarian species (86–184 bp). Benchmarking Universal Single-Copy Orthologs (Simão et al. 2015) analysis showed 74% complete and 7% fragmented conserved eukaryotic genes (80% total), supporting a robust KR01 assembly and annotation.

**Table 1.** Comparison of Genome Features of Available Rhizaria and Other Photosynthetic Species.

	KR01	*Bigelowiella natans*	*Plasmodiophora brassicae*	*Reticulomyxa filosa*	*Phaeodactylum tricornutum*	*Guillardia theta*	*Chlamydomonas reinhardtii*	*Arabidopsis thaliana*
**Genome size (Mb)**	707	95	24	320	27	87	121	140
**GC content (%)**	44	45	59	35	49	53	64	36
**Protein-coding genes**	32,361	21,708	9,730	40,443	10,402	24,840	15,143	26,341
**Genes with introns (%)**	89	86	78	70	47	80	92	79
**Mean intron length (bp)**	882	184	60	86	123	110	373	164
**Mean exons per gene**	10	9	5	3	2	6	8	5

OGF analysis identified 7,374 OGFs in the common ancestor of Rhizaria (fig. 1*D*). The founding lineage of photosynthetic *Paulinella* gained 4,211 novel OGFs that are enriched in signal transduction functions (supplementary table S5, Supplementary Material online), including calcium-binding messengers and serine threonine-protein kinase/phosphatases (supplementary table S6, Supplementary Material online). A total of 98 and 50 well-supported HGT and EGT events (i.e., including expanded gene families), respectively, were identified in a phylogenomic analysis of the predicted KR01 gene models (e.g., supplementary fig. S1, Supplementary Material online). Analysis of the number and putative functions of these EGT-derived genes (supplementary table S7, Supplementary Material online) is consistent with a previous analysis of *P. chromatophora* transcriptome data that indicated a greater role for foreign gene acquisition than EGT in the evolution of this lineage (Singer et al. 2017).

### Chromatophore-Targeted Proteins

The bona fide organelle demarcation for the chromatophore is supported by the finding of two pathways for protein import into this compartment. The first is for long proteins (>268 amino acids in length) that contain a distinctive chromatophore transit peptide (crTP) that is ∼200 aa in length (Singer et al. 2017). Small proteins (<90 aa in length) lack a crTP and appear to rely on the secretory pathway for import (Nowack and Grossman 2012). Use of a hidden Markov model (HMM; Singer et al. 2017) and manual search identified 291 proteins in KR01 that contain a crTP (fig. 1*E*). These protein functions are enriched in genetic information processing: for example, DNA replication and repair, transcription, translation, and regulation (fig. 1*F* and supplementary table S8, Supplementary Material online) with a gene encoding DNA polymerase I-like (*polA*), ATP-dependent helicase (*hrp*B), and NAD-dependent DNA ligase (*lig*A) that are of prokaryotic, eukaryotic, or unclear origins. Both *polA* and *ligA* fill gaps in chromatophore-encoded processes and must therefore be imported into the organelle to restore DNA replication and repair functions. Two genes encoding an ATP-dependent DNA helicase (*recG*, involved in recombination and repair) and a CCA tRNA nucleotidyltransferase (involved in tRNA maturation; supplementary fig. S1*A*, Supplementary Material online) are of α-cyanobacterial provenance. These results demonstrate that chromatophore DNA replication and repair is likely under host (nuclear genome) control vis-à-vis provision of Pol I (encoded by *polA*) and DNA ligase but includes both prokaryotic (non α-cyanobacterial), chromatophore, and eukaryotic contributions.

We found 208 long protein import candidates shared between KR01 and *P. chromatophora* (fig. 1*G*). Barring independent crTP evolution, these represent the ancestral set of chromatophore-targeted proteins in photosynthetic *Paulinella* species that split ∼60 Ma (Lhee et al. 2019). Of this shared set, 135 proteins are assigned to a functional category with the largest being genetic information processing (supplementary fig. S2*A*, Supplementary Material online), consistent with early host control over the genetic system of the endosymbiont. A total of 83/291 chromatophore-targeted proteins are unique to KR01 and evolved after the basal split of these species, indicating a dynamic and divergent path for endosymbiont integration, even in the context of two closely related species. Functional annotation of the KR01-specific gene set showed diverse metabolic functions, similar to that of 208 shared set (supplementary fig. S2*B*, Supplementary Material online).

Only 11 of the KR01 long protein import candidates are clearly of α-cyanobacterial origin (i.e., via EGT), none of which is involved in photosynthesis or light acclimation. The EGT-derived proteins involved in photosynthesis (e.g., high light-inducible [*hli*] genes, *psaE*, *psaK*) are small in size and do not require a crTP for import (Nowack and Grossman 2012). Of the 11 EGT candidates, nine are among the 208 shared chromatophore-targeted proteins. Only seven of the import candidates are of unambiguous bacterial origin and likely have been acquired via HGT and three of these are among the 208 shared import candidates. The majority (273) of KR01 import candidates are of host or unidentified origin (i.e., lack a significant [*e*-value cutoff ≤1e-10] BLAST hit to the nr database).

### Transcriptome Analysis

**Fig. 2.**
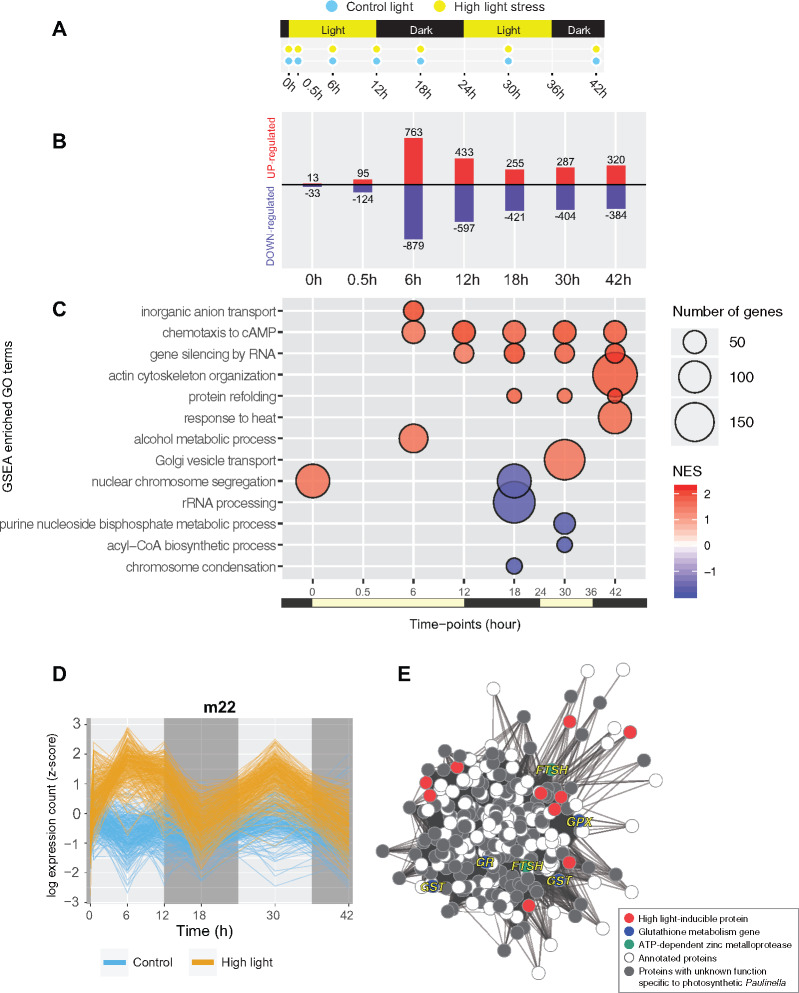
Transcriptome analysis of KR01. (*A*) Experimental design for the RNA-Seq data generation. KR01 cells were exposed to control light and high light stress conditions. Various time points (0, 0.5, 6, 12, 18, 30, and 42 h) under a 12-h/12-h light-dark condition were selected for analysis. (*B*) The number of DEGs under high light stress condition for each time point using DESeq2 (*P* value <0.05 and |log_2_ [fold change]| > 1). (*C*) Enriched GO terms for each time point using GSEA. A positive normalized enrichment score (NES) indicates that a gene set is enriched in the list of genes upregulated, whereas a negative NES indicates that the gene set is enriched in the list of downregulated genes. The size of the circle indicates number of genes in the GO term. (*D*) Individual gene expression patterns in module 22 under control (blue lines) and high light stress (yellow lines) conditions. (*E*) Gene coexpression network of module 22. Each node corresponds to a gene, and a pair of nodes is connected with an edge if there is a coexpression similarity between them. Annotation of a subset of the nodes is shown.

Integration of photosynthetic organelle functions into the diurnal cycle is a major adaptation in novel photosynthetic lineages (Coelho et al. 2013). In addition, the ability of the photosystems to deal with excess light energy that can produce oxidative stress (e.g., reactive oxygen species) (Bratt et al. 2016) provides another key challenge that must be met by lineages such as *Paulinella*. This amoeba lineage is commonly found on the surface of leaf litter in nearshore aquatic environments (Melkonian and Mollenhauer 2005) and therefore encounters fluctuating light levels in a predominantly shaded environment. To address these issues and elucidate the high light stress response in KR01, we generated transcriptome data over the diurnal cycle under a control (10 μmol photons m^−2^ s^−1^) and a high light stress (120 μmol photons m^−2^ s^−1^) condition (fig. 2*A*). The high light stress level used here is quite low when compared with most algae that can withstand three to four times this amount but reflects previous culture work done with *P. chromatophora* (and KR01) that shows growth rates (normally, 5–7-day doubling time) to be reduced above 60 μmol photons m^−2^ s^−1^ and loss of cells to occur after 3 weeks under 120 μmol photons m^−2^ s^−1^ (Zhang et al. 2017).

Under our experimental design, principal component analysis and a heatmap constructed using sample-to-sample distances (supplementary fig. S3, Supplementary Material online) supported the existence of a consistent signal within each set of replicates and strong differentiation between the two treatments. Analysis of differentially expressed genes (DEGs) for each time point (supplementary table S9, Supplementary Material online) showed that 0 h had the smallest number of DEGs (46) and 6 h the largest (1,642) (fig. 2*B*), with lowered gene expression 12 h onward. To explore the biological function of DEGs, gene ontology (GO) enrichment analysis was done using gene set enrichment analysis (GSEA) (fig. 2*C*) and showed that most of the enriched GO terms were found after 6 h (supplementary fig. S4, Supplementary Material online).

#### Gene Coexpression Analysis

We used gene coexpression analysis to investigate the RNA-Seq data. Gene coexpression networks can be used to associate genes of unknown function with biological processes, to prioritize candidate genes, or to identify transcriptional regulatory programs (van Dam et al. 2017). We found 71 distinct modules of gene coexpression (supplementary fig. S5, Supplementary Material online). Examples include module 1 (m1) enriched in actin-related GO terms, chemotaxis, and signaling, whereas “RNA processing” was enriched in m4, “potassium ion transport” in m30, and “DNA replication” in m2 (supplementary table S10, Supplementary Material online). We calculated module enrichment in terms of proteins with the features crTP/HGT/diurnal expression and DEGs at each time point (|log_2_ [fold change]| > 1) (supplementary table S11, Supplementary Material online). Of particular interest was the *hli* (Komenda and Sobotka 2016) gene cluster that includes components of the high light response “toolkit” in KR01. Among 34 *hli* genes with assigned modules, 13 were in m22 showing upregulation under high light (supplementary table S12, Supplementary Material online). Besides the 13 HLI proteins (red), the m22 coexpression network contained four glutathione-related proteins (blue), as well as a variety of dark proteins (gray) (fig. 2*D* and *E*; supplementary table S13, Supplementary Material online). Other genes in module m22 include ATP-dependent zinc metalloprotease (*ftsH*), which has a variety of functions (e.g., major thylakoid membrane protease) and is involved in the repair of the D1 protein in PSII under light induced damage (Shao et al. 2018). Nuclear-encoded eukaryotic *ftsH* exhibited the same expression pattern as *hli* genes, suggesting that FtsH could be targeted to the chromatophore and is involved in D1 repair.

In addition, we found that components of the proteosome were generally upregulated under high light, although expression differences were not significant using DESeq2 (|log_2_ [fold change]| > 1) (supplementary fig. S6*A* and table S14, Supplementary Material online). Coexpression network analysis showed that cytosolic ribosomal protein genes were downregulated under high light stress, suggesting growth inhibition (supplementary fig. S6*B* and table S15, Supplementary Material online). The results of this analysis provide the basis for selecting candidate genes for downstream analysis of the high light stress response in KR01.

#### Diurnally Rhythmic Genes in KR01 and Archaeplastida

**Fig. 3.**
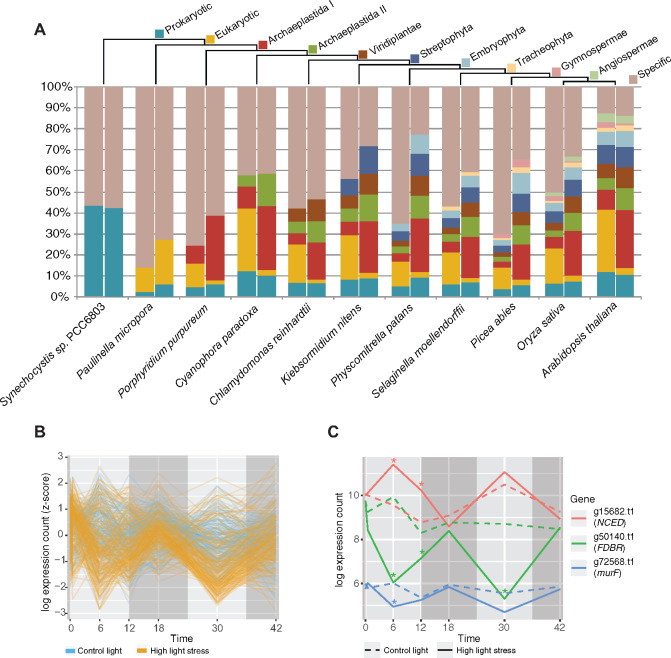
Diurnal gene expression analysis and expression pattern of genes that contain crTP in KR01. (*A*) Percentage OGFs sorted according to their relative age (tree drawn on the top) for each species. There are two bars for each species; the left bar is based on whole gene sets and the right bar is based on only diurnally rhythmic genes. (*B*) Expression patterns of 228 HMM-predicted chromatophore-targeted proteins in KR01 under control and high light stress conditions. (*C*) Expression pattern of three genes that contain a crTP, *murF*, *FDBR*, and *NCED*. Asterisks (*) indicate significant differences in expression count by DESeq2 (*P* value <0.05 and |log_2_ [fold change]| > 1).

Comparison of 2,435 DEGs (|log_2_ [fold change]| > 1 between any two time points) and 3,853 rhythmic genes identified using the Jonckheere–Terpstra–Kendall (JTK) algorithm (Hughes et al. 2010), resulted in an overlap of 1,354 KR01 genes that are both DEGs and exhibit rhythmic behavior (supplementary fig. S7 and table S16, Supplementary Material online). The largest number of diurnally regulated genes was expressed at 18 h (night, 393 genes) and 6 h (day, 375 genes) and overall was enriched with the category “carboxylic acid biosynthetic process” (supplementary table S17, Supplementary Material online). To gain insights into the origin and evolution of diurnally regulated genes in KR01 and in Archaeplastida, OGFs were sorted according to their relative age (fig. 3*A*). A previous study (Ferrari et al. 2019) demonstrated that despite large phylogenetic distances and dramatic differences in morphology and lifestyle, diurnally rhythmic genes in Archaeplastida species are conserved across lineages; that is, many diurnally rhythmic genes were inherited from the common ancestor of the first algae (the Archaeplastida I component), likely due to selection to adapt to the diurnal light-dark cycle. When comparing KR01 and the Archaeplastida, diurnally rhythmic genes show a vastly different pattern even though they share a large fraction of genes of eukaryotic origin.

**Fig. 4.**
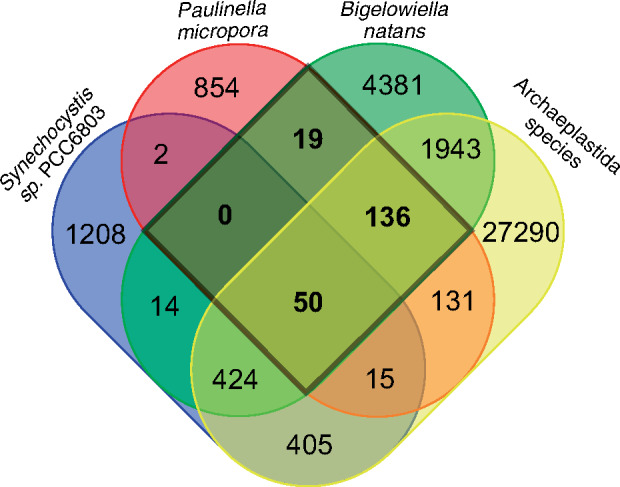
Venn diagram of OGFs containing diurnally rhythmic genes shared by *Paulinella micropora*, *Bigellowiella natans*, *Synechocystis* sp., and Archaeplastida species.

When we inspect the KR01 set of diurnally regulated genes, most of them that are present also in both *Synechocystis* sp. and Archaeplastida are functionally annotated (87/92 genes or 94.6%) and enriched with “chloroplast” GO terms (supplementary table S18, Supplementary Material online). Diurnal KR01 genes shared only with Archaeplastida (365 genes) were enriched with “rRNA processing.” In contrast, of the much larger independently derived diurnal gene set in KR01 only 16.8% (or 151/897 genes) has a functional annotation. We expect that some of the original diurnal genes were integrated into the photosynthetic lifestyle but the KR01 number is small (4%, 1,354/32,361) when compared with Archaeplastida, in which single-celled algae have a mean of 49.3% rhythmic genes. Land plants have a mean of 31.8% rhythmic genes (Ferrari et al. 2019). *Bigellowiella natans*, another photosynthetic rhizarian with a green alga derived secondary plastid has 35.7% diurnally rhythmic genes (Suzuki et al. 2016). When diurnally rhythmic OGFs were compared, including *B. natans*, only 9% (19/205) were shared exclusively between KR01 and *B. natans* (fig. 4). This implies that Rhizaria-specific genes are a minor component of diurnal regulation in KR01. Our results underline the importance of the *Paulinella* model vis-à-vis the evolution of an essentially novel set of diurnally regulated genes that respond to light stress (fig. 2*D* and *E*) and the light-dark cycle (fig. 3*A*).

#### *Antioxidant Defense in Photosynthetic* Paulinella

Photosynthetic species have developed several strategies to cope with oxidative stress. The major components of the antioxidant system in plants include ascorbic acid, glutathione, and tocochromanols. Genes encoding antioxidant enzymes also come into play, such as catalase (*cat*), superoxide dismutase (*sod*), and peroxiredoxins (*prx*), among others (Szymańska et al. 2017). In cyanobacteria, genes encoding flavodiiron proteins (*flv*), an orange carotenoid protein (*ocp*), and Hlips (*hli*) are upregulated to protect against excess light energy (Muramatsu and Hihara 2012). In the case of KR01*, hli* (supplementary table S12, Supplementary Material online), *prx* (supplementary table S19, Supplementary Material online), and glutathione-related genes (supplementary table S20, Supplementary Material online) exhibit differential expression under high light stress condition. It is not clear whether ascorbic acid and tocochromanols are present in KR01 because we could not find all the genes involved in their biosynthesis. Antioxidant enzyme genes such as *cat*, *flv*, and *ocp* were also not found in our data set. However, *sod* was present, although its expression was not significantly altered under high light stress (supplementary table S21, Supplementary Material online).

Expansion of the *hli* gene family and upregulation under high light stress suggests that these genes play an important role in the high light stress response in KR01. To identify a potential high light regulatory motif in KR01, we searched 500-bp upstream and downstream of each *hli* gene in the genome data (Materials and Methods). Many *hli* genes were in pairs and some had not previously been functionally annotated (supplementary fig. S8, Supplementary Material online). Conserved motifs were discovered (supplementary fig. S9, Supplementary Material online), suggesting a potential regulatory function for these sequences.

Glutathione metabolism genes appear to play an important role under high light stress because four genes were in m22 and exhibited the same expression pattern as *hli* genes and four other genes had crTPs (supplementary table S20, Supplementary Material online). Glutathione is an abundant metabolite in plants that scavenges OH and singlet oxygen, may protect enzyme thiol groups, and is also involved in signal transduction (Foyer and Noctor 2005). After glutathione is biosynthesized by GSH1 (glutamate–cysteine ligase) and GSH2 (glutathione synthetase), it can participate in the detoxification of reactive electrophilic compounds by GPX (glutathione peroxidase) and GST (glutathione S-transferase) (Nutricati et al. 2006; Rouhier 2010) (supplementary fig. S10, Supplementary Material online). Glutathione reductase (GR) reduces glutathione disulfide to glutathione using NADPH as an electron donor and the overexpression of GR leads to increases in antioxidant capacity (Foyer et al. 1995). When we searched for glutathione metabolism genes in the KR01 data, one was identified (GSH1) in the chromatophore genome, and other genes were nuclear encoded: that is, one GSH2, two GR, four GPX, and 14 GSTs. We could distinguish two different expression patterns among glutathione metabolism genes. The first was for genes encoding GR, GPX, and GST that shared the same expression pattern as *hli* in m22, whereby only *GST* and *GPX* showed a significant expression difference (|log_2_ [fold change]| > 1) under high light stress. The second pattern included genes that contain a crTP and were downregulated, whereby only *GR* showed a significant expression difference. In *Arabidopsis thaliana*, *GR*, *GST*, and *GPX* are induced under high light stress (Kimura et al. 2003). As in the plant system (Gill et al. 2013), some GST and GR proteins were targeted to the chromatophore in KR01. This suggests that an analogous system for mitigating the negative impacts of high light stress has evolved in the *Paulinella* primary endosymbiosis, although chromatophore-destined proteins show a different expression pattern under high light stress than described in plants. Further study is needed to understand the glutathione-based antioxidant response in KR01.

#### *Cryptochrome/Photolyase Family in Photosynthetic* Paulinella

The cryptochrome/photolyase family (CPF) comprises flavoproteins with similar structures that display a variety of light-dependent functions. This family encompasses photolyases, blue-light-activated enzymes that repair ultraviolet-light induced DNA damage, and cryptochromes that regulate a variety of functions (e.g., circadian rhythm and developmental signals) in plants and animals (Fortunato et al. 2015). Even though CPFs are present in nonphotosynthetic organisms, in photoautotrophs, CPFs regulate light acclimation and adaptation processes, suggesting that specific CPFs may have coevolved with regulatory signals that control plastid function. A previous study (Zhang et al. 2017) of *P. chromatophora* showed that nuclear-encoded *hli* genes are induced during exposure to blue light (2 μmol photons m^−2^ s^−1^) and these authors found two cryptochrome (CRY)-like encoding genes in the transcriptome assembly of this species. We identified nine CPFs in the genome of KR01, including partial and pseudogene copies (supplementary table S22 and fig. S11, Supplementary Material online). Interestingly, four of these genes show a diurnal expression pattern and two contain a crTP. Moreover, three CPF-encoding genes show differential expression under high light stress. In *A. thaliana*, CPFs play a key role in circadian clock regulation (Jarillo et al. 2001) and the synthesis of various photoprotective components (Kleine et al. 2007). In cyanobacteria, Cry-DASH regulates photosynthetic activity and PSII repair (Vass et al. 2013). Cry-DASH is localized to the chloroplast in *A. thaliana* (Kleine et al. 2003). Using these studies as a guide, our finding that CPFs in KR01 are differentially regulated under high light stress and show a diurnal expression pattern, suggests these proteins are important players in light regulation of chromatophore functions in *Paulinella*.

#### Differential Expression of Genes Encoding Chromatophore Transit Peptide

More than 3,000 proteins encoded by the nuclear genome are imported into the chloroplast and play important roles in organelle function (Richly et al. 2003). We investigated the expression of genes encoding crTPs in KR01 and found that most are downregulated under high light stress (fig. 3*B*). Examples include the HGT-derived genes *murF* (UDP-N-acetylmuramoyl-tripeptide: D-Ala-D-Ala ligase) (Heijenoort 2001) that provides the missing step in peptidoglycan biosynthesis in the chromatophore and ferredoxin-dependent bilin reductase (FDBR) (fig. 3*C*). FDBRs are involved in the breakdown of heme to different bilins that act as chromophores for light-harvesting (phycobiliproteins) and/or light-sensing functions (phytochromes; Rockwell and Lagarias 2017). It is interesting that bilin biosynthesis is present in all oxygenic photosynthetic lineages, regardless of the existence of phytochromes or phycobiliproteins (Rockwell et al. 2014). We found two FDBR-encoding genes in KR01. The first, *pcyA*, is encoded on the chromatophore genome and is of cyanobacterial origin, whereas the second, the product of gene g50140.t1, is nuclear encoded and targeted to the chromatophore (supplementary fig. S12, Supplementary Material online). Phylogenetic analysis of FDBRs demonstrates the independent derivation of these proteins in KR01 (supplementary fig. S13, Supplementary Material online). The complete pathway for the biosynthesis of phycocyanobilin is still present on the chromatophore genome, however, downregulation of the chromatophore-targeted *FDBR* under high light stress suggests that bilin biosynthesis in KR01 may be under nuclear regulation.

In contrast to downregulated genes, there were several chromatophore-targeted proteins that were significantly upregulated under high light. One is 9-cis-epoxycarotenoid dioxygenase (NCED) that catalyzes the first step of abscisic-acid (ABA) biosynthesis (fig. 3*C*). ABA is a stress-related signaling molecule reported in all kingdoms of life except Archaea (Hauser et al. 2011). In *A. thaliana*, NCED is the rate-limiting step in the ABA biosynthesis pathway. Moreover, *NCED* is induced by stress and targeted to the chloroplast (Xiong and Zhu 2003). In *Chlamydomonas reinhardtti*, ABA protects the PSII complex from photo-induced damage (Saradhi et al. 2000). These studies and our result point to the possibility that ABA is a stress response mechanism in KR01.

### Analysis of Phage and Viral DNA in the KR01 Genome

A recent analysis of the genome of the closely related strain *P. micropora* MYN1 (hereafter, MYN1) led to the hypothesis that large double-stranded DNA (dsDNA) viruses may have facilitated early gene transfer events to the amoeba nucleus, allowing rapid integration of the chromatophore (Matsuo et al. 2019). We previously documented the precipitous loss of chromatophore genes soon after organelle origin ∼124 Ma (Lhee et al. 2019), whereby at the basal split of extant photosynthetic *Paulinella* species, 1,620 OGFs, or 65% of the gene inventory, had been deleted leaving behind 882 OGFs in the chromatophore. Gene loss slowed down thereafter with only 40–44 OGFs being lost during the ensuing 60 My. We hypothesized that Muller’s ratchet (Muller 1932; Felsenstein 1974; Moran 1996), resulting from reduction in population size and random genetic drift, typical of asexually reproducing populations (in this case, permanent endosymbionts) led to loss-of-function mutations and thereby, gene deletion or transfer to the nucleus via EGT (e.g., due to chance organelle DNA integration into the nuclear genome; Stegemann et al. 2003). In contrast to this model of chromatophore genome reduction driven by neutral processes, Matsuo et al. proposed that large DNA viruses containing Maverick/Polinton-type transposons may have infected the amoeba lineage at the time of endosymbiosis and acted as vectors of both EGT and HGT to the nucleus (Matsuo et al. 2019). Of course, both of these processes could have occurred during the early phases of chromatophore integration. To test the hypothesis of large dsDNA virus-mediated EGT/HGT, we mapped an extensive library of *P. chromatophora* Illumina short reads we had previously generated (Nowack et al. 2016) to MYN1 Scaffold1104 that is enriched in Maverick/Polinton-type transposons (see fig. 3*A* in Matsuo et al. 2019). The prediction was that if these selfish genetic elements had played a critical role in photosynthetic *Paulinella* genome evolution, then they should also be present in the genome of *P. chromatophora*, as well as in KR01.

This analysis showed that only 1.75% and 3.12% of the bases of the dsDNA virus regions in the MYN1 genome Scaffold1104 were covered by *P. chromatophora* mapped reads, using global and local alignment modes, respectively (supplementary fig. S14*A*, Supplementary Material online). This stands in stark contrast to the non-dsDNA viral regions that had 50.45% and 55.12% of bases covered by mapped reads (using global and local alignment modes, respectively). No scaffolds from the *P. chromatophora* genome were found to have similarity to the dsDNA viral regions of Scaffold1104. Analysis of the MYN1 genome identified 28.6 Mb of sequences with similarity to the putative DNA viral sequences described in that article. However, only 0.25% and 0.98% of the bases of these regions are covered by *P. chromatophora* mapped reads using global and local alignment modes, respectively. These results suggest that the dsDNA viral regions observed in MYN1 are absent or poorly represented in the genome of *P. chromatophora*. There are however large regions of high similarity between the dsDNA viral regions in KR01 and MYN1 (supplementary fig. S14*B*, Supplementary Material online), as would be expected for these closely related strains (MYN1 is an isolate of strain FK01 [a close relative of KR01]) that share 99.85% sequence identify over their chromatophore genomes (Lhee et al. 2017). These data are more consistent with a recent invasion by large dsDNA viruses in the KR01/MYN1 lineage, rather than a more ancient origin in the photosynthetic *Paulinella* ancestor. Alternatively, these transposons may have been lost in the *P. chromatophora* genome, although this seems less likely given this genome has expanded to a massive size of ∼10 Gb (Nowack et al. 2016) when compared with KR01 or MYN1. The generation of additional *Paulinella* genomes will help resolve this important issue. What we can however conclude with some confidence at this juncture is that EGT and HGT were not the dominant players in chromatophore domestication in terms of gene numbers, with only about 50 and 98 events, respectively, going to fixation in KR01. Far more significant (∼94% of crTP-containing proteins) was the redeployment of existing host genetic resources via protein retargeting to the organelle to rescue existing pathways and ensure the maintenance of organelle DNA.

## Conclusions

The origin and diversification of photosynthesis in eukaryotes has been intensely researched (Keeling 2010; Price et al. 2012; Cenci et al. 2017) and provided the foundation for understanding the evolution of this complex trait and for generating “green energy” sources (Li-Beisson et al. 2019; Anto et al. 2020). Despite this effort, it is clear that many aspects of the earlier stages of primary endosymbiosis are beyond the reach of ancestral state reconstruction using Archaeplastida species. For this reason, the *Paulinella* model has proven invaluable because of the many insights it has provided into plastid integration (Nowack et al. 2008; Bhattacharya et al. 2012; Lhee et al. 2019), including the evolution of novel protein import systems (Nowack and Grossman 2012; Singer et al. 2017), the impact of E/HGT on organelle functions (Nowack et al. 2016), composition of the ancestral plastid proteome, the evolution of diurnal rhythmicity, novel stress response pathways, and the role of dark genes in underpinning this evolutionary leap. The high-quality genome assembly and results described here lays the foundation for additional functional analyses and testing of KR01 novel gene functions in both native and heterologous systems to harness adaptations that arose in this unique amoeba lineage.

## Materials and Methods

### Sampling, Isolation, and DNA/RNA Extraction

KR01 cells were isolated from a natural freshwater sample collected on August 18, 2009, from Mangae Jeosuji (Reservoir), Chungnam Province, South Korea (Lhee et al. 2017). An axenic culture of *P. micropora* KR01 was produced following an existing method (Nowack et al. 2016). The DNeasy Plant Mini Kit (Qiagen, Santa Clarita, CA) was used for the DNA extraction and RNA was extracted using the TRI reagent (Molecular Research Center, Cincinnati, OH).

### Sequencing 

To generate the genome data, large fragments of KR01 DNA from the axenic culture were used for Illumina HiSeq 2500 sequencing (supplementary table S1, Supplementary Material online). A bacteria-reduced culture was used for PacBio RS II and HiSeq 2500 sequencing. For transcriptome sequencing, control and high light stress conditions (control = 10 µmol photons m^−2 ^sec^−1^; stress = 120 µmol photons m^−2 ^sec^−1^; see Zhang et al. [2017]) over a 12/12-h light/dark cycle and temperature stress conditions (control = 24 °C; stress = 4 °C, 38 °C) were sequenced using the Illumina Truseq stranded mRNA library prep kit (supplementary table S2, Supplementary Material online). All sequencing was done by DNA Link Inc. (Seoul, Korea). Detailed sequencing output are provided in the supplementary tables S1 and S2, Supplementary Material online.

### De Novo Genome Assembly and Assessment

We generated a total of 149 Gb of single-molecule real-time sequences using the P6-C4 sequence chemistry with an average subread length of 7.9 kb. De novo assembly was done using the FALCON-Unzip assembler (Chin et al. 2016) with filtered subreads. The length cutoff option was specified based on the subread N50 value of 12 kb. The assembly had a contig N50 = 147 kb for the phased diploid assembly that was “polished” using Quiver (Chin et al. 2013). We performed error correction using BWA (Li and Durbin 2009) and GATK (DePristo et al. 2011) with Illumina HiSeq reads to improve the quality of the assembly. Following this procedure, “contaminant” contigs were removed after identification by searching the NCBI bacteria database using BlastN (Camacho et al. 2009). A repeat library of the KR01 genome was constructed using RepeatModeler (Smit and Hubley 2008–2015) with masking using RepeatMasker (Smit et al. 2013–2015).

### Gene Prediction and Annotation

Gene prediction was done with BRAKER2 (Stanke et al. 2006; Hoff et al. 2016). The following data were used for hints: RNA-Seq data (high and control light condition 0, 0.5, 6, 12, 18, 30, and 42 h), de novo-assembled RNA-Seq data using Trinity (Grabherr et al. 2011), and *P. chromatophora* CCAC0185 protein data (Nowack et al. 2016). To filter out genes with low RNA-Seq read coverage, axenic RNA-Seq data (different temperature: 24, 4, and 38 °C) were aligned to the genome assembly using the STAR tool under default parameters (Dobin et al. 2013). Predicted genes with average coverage <1 were removed. Among the different transcripts from same gene, we choose the first transcript (named “.t1” in the gtf output file) and removed the remainder. Predicted genes were annotated using eggNOG-Mapper v2 (Huerta-Cepas et al. 2017) with the EggNOG database (Huerta-Cepas et al. 2019). In addition, individual proteins were searched against locally installed NCBI nr database (February 1, 2019) using MMseqs2 (Steinegger and Söding 2017) with a cutoff *e*-value = 1e^−05^. We assessed genome completeness, vis-à-vis the predicted genes, using Benchmarking Universal Single-Copy Orthologs (Simão et al. 2015).

### Orthologous Gene Families Analysis

Protein sequences from *A. thaliana* (GCA_000001735.2), *Dictyostelium discoideum* (GCA_000004695.1), *Homo sapiens* (GCA_000001405.28), *Naegleria gruberi* (GCA_000004985.1), *Paramecium tetraurelia* (GCA_000165425.1), *Phaeodactylum tricornutum* (GCA_000150955.2), *B. natans* (GCA_000320545.1), *Plasmodiophora brassicae* (GCA_001049375.1), and *Reticulomyxa filosa* (GCA_000512085.1) were retrieved from NCBI. Protein sequences from *P. chromatophora* CCAC0185 were retrieved from Singer et al. (2017). Orthologous gene families (OGFs) of proteins were clustered using OrthoFinder (Emms and Kelly 2015). Clusters of Orthologous Groups (COG) function of OGF were annotated using eggNOG-Mapper result from rhizarian species. Ancestral OGF numbers with gain and loss were estimated using the Dollo parsimony principle (Csűös 2010). Pfam-domain (El-Gebali et al. 2019) of KR01 genes was searched using HMMER (Eddy 2011). COG function enrichment of OGFs were done by gProfiler (Raudvere et al. 2019).

### Analysis of Horizontal and EGT

Each predicted KR01 protein was used in a BlastP query against an in-house database composed of NCBI RefSeq v.95 proteins with the addition of available algal and protist genome and transcriptome data from dbEST, TBestDB, the JGI Genome Portal (https://genome.jgi.doe.gov; last accessed August 19, 2020) and the Moore Microbial Eukaryote Transcriptome Sequencing Project (Keeling et al. 2014). This database was partitioned into four subsets based on taxonomic provenance: Bacteria, Opisthokonta, remaining nonbacterial or opisthokont taxa, and the Moore Microbial Eukaryote Transcriptome Sequencing Project database. Each subset was searched against independently (BlastP [Camacho et al. 2009], *e*-value ≤1e^−10^) using the KR01 proteins and the top 2,000 hits from each search were saved, combined, and sorted by bitscore. The sorted list was parsed such that a taxonomically broad selection of top hits was retained, and the associated proteins were aligned together with the query sequence using MAFFT (Katoh and Standley 2013). Maximum-likelihood phylogenetic trees were constructed using IQTREE (Nguyen et al. 2015) after automatic model selection with nodal support tested via 2,000 ultrafast phylogenetic bootstraps (Minh et al. 2013).

Phylogenetic trees with evidence of putative HGT or EGT events were identified using the PhySortR package (Stephens et al. 2016). Trees with clades composed of *P. micropora* and Bacteria sequences (min.support = 70, min.prop.target = 0.7, clades.sorted = “E, NE,” and clade.exclusivity = 0.95) were retrieved for manual filtering; clades with *Paulinella* sequences from other isolates were also retrieved. Trees were discarded if they had <10 leaves, <5 Bacterial taxa, or multiple *Paulinella* sequences not in the same clade. Eukarya/Archaea taxa in the tree had to be separated from KR01 by well-supported nodes. Trees were discarded if their alignments lacked significant conserved regions or if the *Paulinella* sequences did not align to the conserved regions. Putative EGT events were identified in the same way as HGT events, except only clades composed of cyanobacteria were retrieved, with manual verification of *Paulinella* grouping with α-cyanobacteria.

### In Silico Prediction of KR01 Chromatophore Import Candidates

To computationally identify chromatophore transit peptide (crTP) candidates within the 32,361 translated nuclear transcripts from KR01, we utilized a HMM. Initially, CCAC0185 crTPs were blasted into the KR01 protein database, resulting in the identification of 31 protein hits (*e*-value cutoff = 1e^−20^). Based on a manually curated alignment of these proteins, a HMM profile was built and calibrated for KR01. This profile was used to search for crTPs (*e*-value = 1e^−05^) using the full set of KR01 predicted proteins. The output (228 proteins) represent protein sequences in which the HMM profile was recognized to start between amino acid positions 1 and 50 and in which the encoded mature proteins were >250 amino acids in length. To extend these results, we used each predicted crTP from KR01 as queries against the proteome database of this species to find sequences that may have escaped detection using the HMM approach. This search turned up an additional 59 high-quality candidates, resulting in a final list of 291 crTP-containing proteins from KR01. To determine the putative functions of these chromatophore targeted- protein candidates, we utilized BlastP, GO analysis, and the KEGG database to assign them to functional categories.

### Transcriptomics

#### Experimental Design

Mass cultured cells were isolated using a 10-µm pore size membrane filter and then divided into 50-ml transparent Falcon tubes with fresh DY-V medium. After incubation in the growth chamber for 2–3 days, cells were exposed to two different intensities of white LED light: 10 μmol photons m^−2^ s^−1^ (control) and 120 μmol photons m^−2^ s^−1^ (high light). Different time points (0, 0.5, 6, 12, 18, 30, 42 h) under a 12-h/12-h light/dark cycle were used for this analysis (fig. 2*A*). The conditions corresponding to the seven control light time points are denoted as CL0, CL0.5, CL6, CL12, CL18, CL30, and CL42, whereas the high light (HL) treatments are denoted as HL0, HL0.5, HL6, HL12, HL18, HL30, and HL42. Cells from triplicate cultures at these conditions were harvested using a 10-µm pore size membrane filter, frozen in liquid nitrogen, and stored at −80 °C. RNA was extracted using the TRIzol reagent (Molecular Research Center, Inc.).

#### Differential Gene Expression Analysis

The RNA-Seq reads were trimmed using Trimmomatic (Bolger et al. 2014) and mapped on KR01 gene models using Salmon in quasi-mapping mode (Patro et al. 2017). The raw reads were counted at the gene level and imported into DESeq2 for normalization (Love et al. 2014). A total of 22,361 genes had expression in at least two triplicates from every condition. Significantly DEGs between groups were identified using DESeq2. Only, the genes with Benjamini–Hochberg adjusted *P* value (padj) <0.05 and |log_2_ [fold change]| > 1 were considered for downstream analysis. Principal component analysis and hierarchical clustering of the sample distances were done using the DESeq2 package (Love et al. 2014).

#### Coexpression Networks

To cluster genes into shared expression patterns, the 22,361 genes with expression in at least two triplicates from every condition were used for this analysis. Expression of each condition was averaged and used for WGCNA (Langfelder and Horvath 2008) with option: Pearson, Signed, minimum module size 10, merge height 0.15. WGCNA produced 71 modules with sharing expression patterns, except for module 0 which includes genes that did not cluster. For expression-level visualization of modules, log normalized counts were converted to a *Z*-score. Coexpression network m22 was visualized using Cytoscape v3.1 (Shannon et al. 2003).

#### Enrichment

For the enrichment analysis, a Gene Matrix Transposed file was manually constructed using assigned GO terms and KEGG pathways in the eggNOG-Mapper (Huerta-Cepas et al. 2017, 2019) result. Enrichment analysis of DEGs was done using GSEA (Subramanian et al. 2005). GO terms with a false discovery rate <25% were chosen. For GO term and KEGG pathway enrichment of specific gene sets (e.g., diurnally rhythmic genes), gProfiler (Raudvere et al. 2019) was used (*P* value <0.05). For coexpression module enrichment analysis, Gene Matrix Transposed file was manually constructed with assigned module data and gProfiler (Raudvere et al. 2019) was used (*P* value <0.05). GO terms were summarized by REVIGO (Supek et al. 2011).

#### Motif Screening

Putative promoter sequences (500-bp upstream from the start codon) were extracted from the genome sequence of KR01 for the *hli* gene set. MEME (v5.0.4) (Bailey and Elkan 1994) was used to screen for motifs, with default parameters except for the following parameters: site distribution and number of motifs = 10.

### Diurnally Rhythmic Genes

Diurnally rhythmic genes in KR01 were identified using the JTK algorithm (*P* value <0.01) using four time points (control light 0, 6, 12, and 18 h) from the transcriptome data (Hughes et al. 2010). We found 1,354 DEGs (|log_2_ [fold change]| > 1 between any two time point) to be JTK-based rhythmic genes. To compare the diurnally rhythmic genes of KR01 with Archaeplastida species, OGFs were sorted using OrthoFinder (Emms and Kelly 2015). We retrieved the publicly available diurnally rhythmic gene expression data (Suzuki et al. 2016; Ferrari et al. 2019). The OGFs were considered diurnally rhythmic for each species if there were least one diurnally rhythmic gene. Because the order of appearance of the clades is well supported (Bhattacharya and Price 2020; Burki et al. 2020), the OGFs could be sorted according to their relative age by finding the oldest clade in the family, from the oldest (i.e., prokaryotic) to youngest (e.g., specific to *A. thaliana*).

## Data Availability

The genome assembly, gene models, expression data, and functional annotation of *Paulinella micropora* KR01 genes are available at the Marine Bioinformation Center, National Marine Biodiversity Institute of Korea (http://www.magic.re.kr/; last accessed August 19, 2020) (raw data: MN00067, MN00096, and MN00126; assembly data: MA000302) and at http://cyanophora.rutgers.edu/P_micropora/ (last accessed August 19, 2020), where the phylogenomic results are also available for download.

## Supplementary Material

Supplementary data are available at *Molecular Biology and Evolution* online.

## Supplementary Material

msaa206_Supplementary_Data

## Notes

The raw data are deposited at GenBank under the BioProject accession PRJNA568118.
