# Supplementary material for: Amoeba Genome Reveals Dominant Host Contribution to Plastid Endosymbiosis
Source: Mol Biol Evol. 2020 Aug 13;38(2):344–57. doi: 10.1093/molbev/msaa206 (PMC7826189; doi:10.1093/molbev/msaa206)
Supplement: msaa206_Supplementary_Data [file msaa206_supplementary_data.zip › msaa206-suppl_data/Supplementary information_final.pdf]

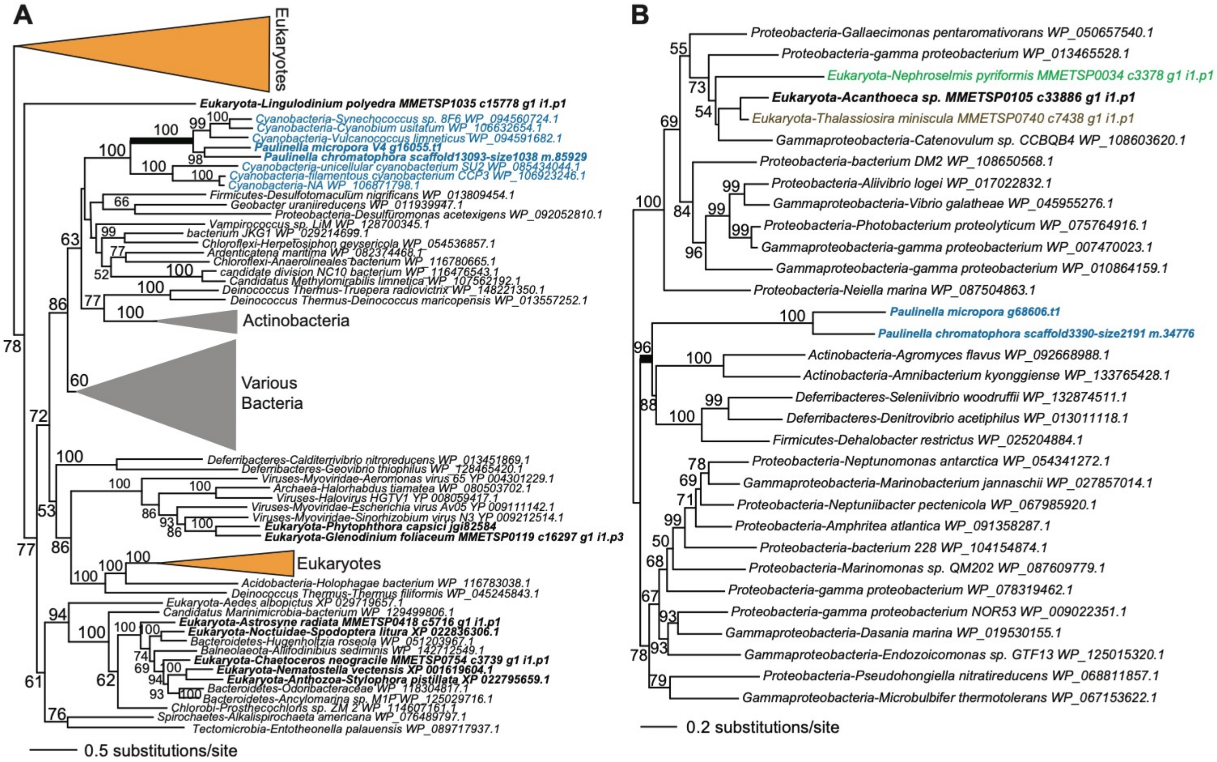

Supplementary fig. S1. Phylogeny of HGT/EGT-derived genes in KR01. Maximum likelihood phylogenetic trees from amino acid alignments of (A) g16055.t1 and (B) g68606.t1.

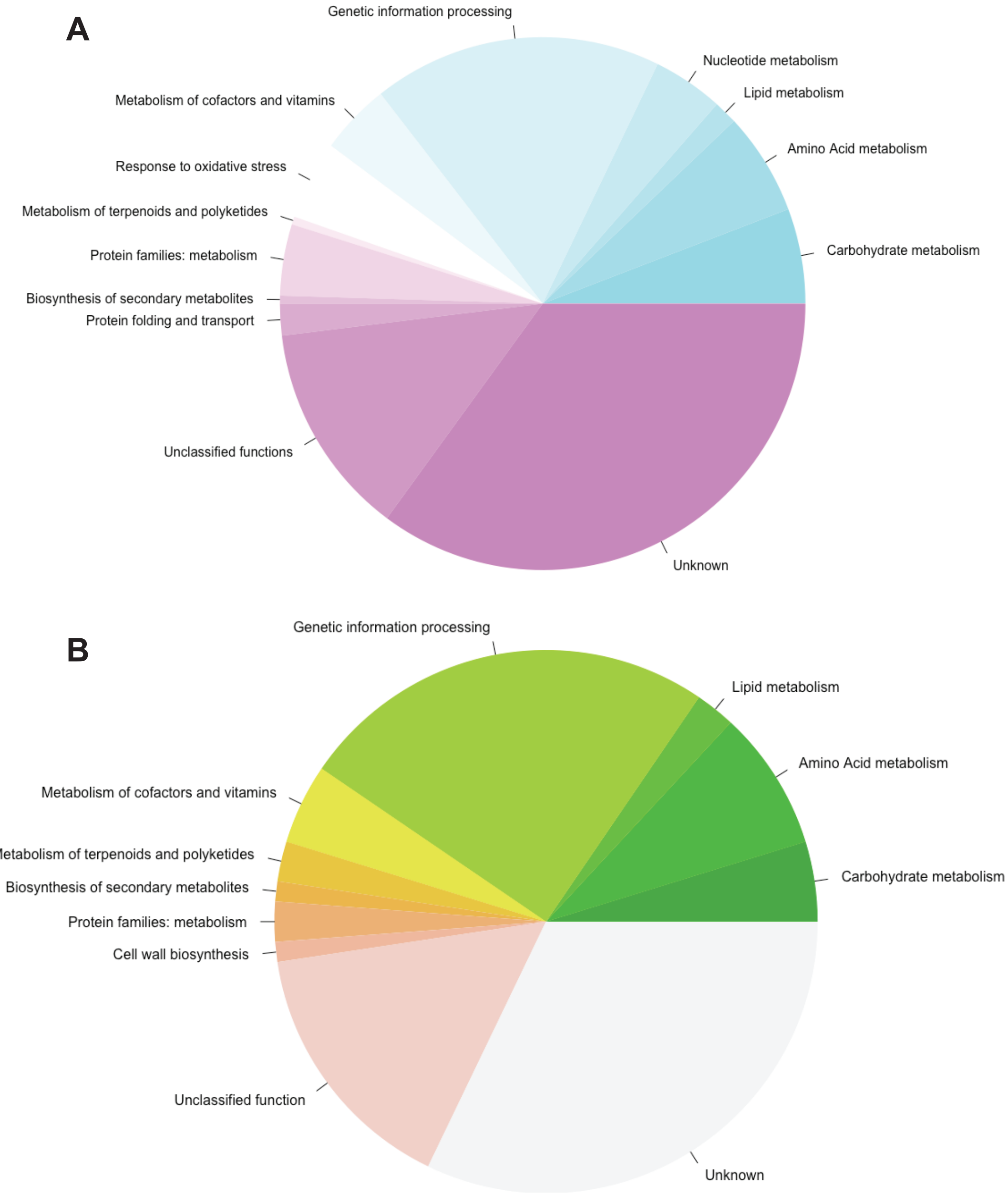

Supplementary fig. S2. (A) Functional classification of 208 import candidates shared by KR01 and *P. chromatophora*. These comprise the putative ancestral import candidates in the *Paulinella* lineage. (B) Functional annotation of the predicted 83 import candidates unique to KR01. Functional annotation was generated using KAAS and categorized based on KEGG functional categories.

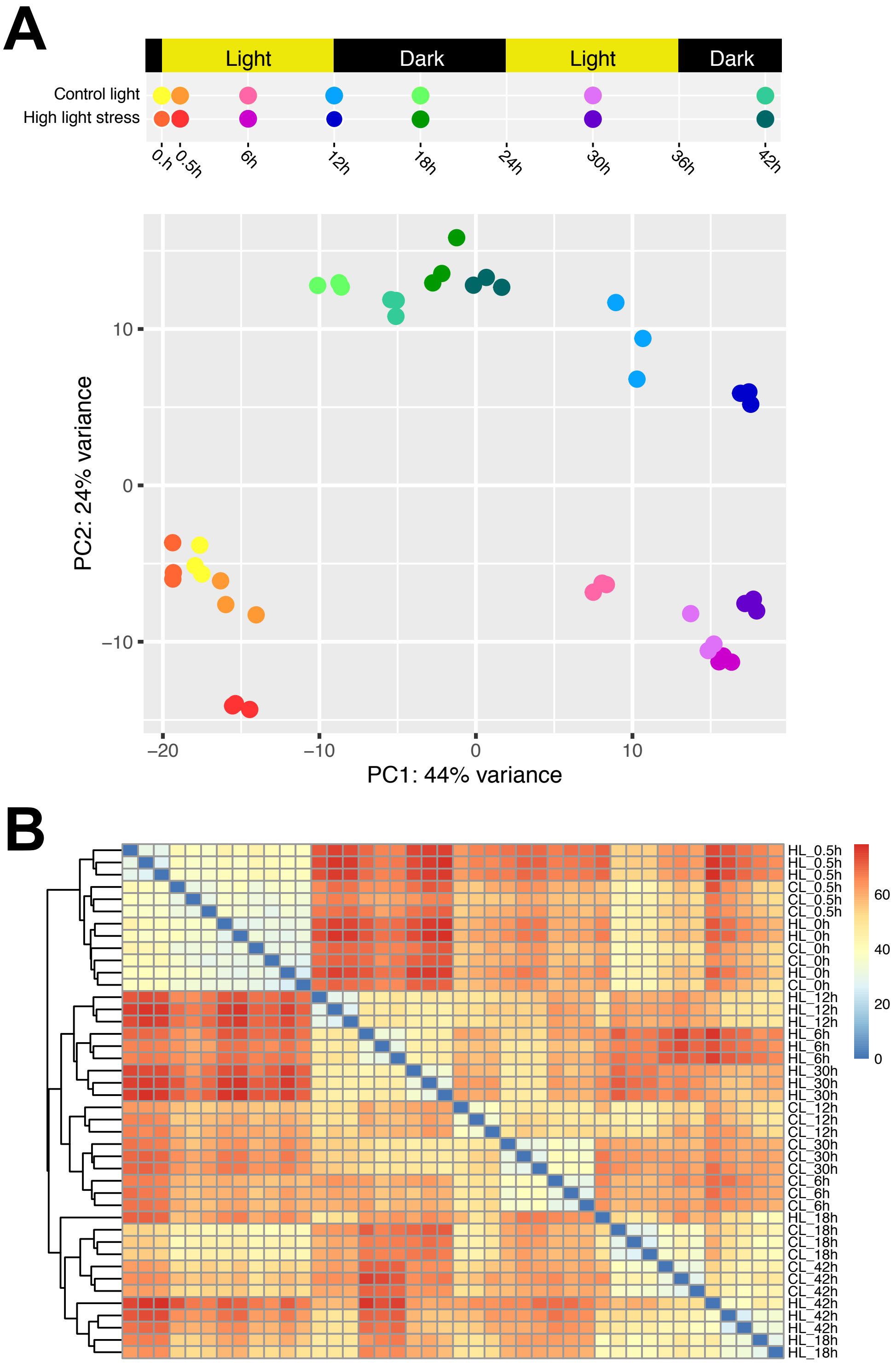

Supplementary fig. S3. (A) Principal component analysis of each conditions using the normalized log count data. Each condition (triplicates) is colored differently. (B) Hierarchical clustering of sample-to-sample distances.

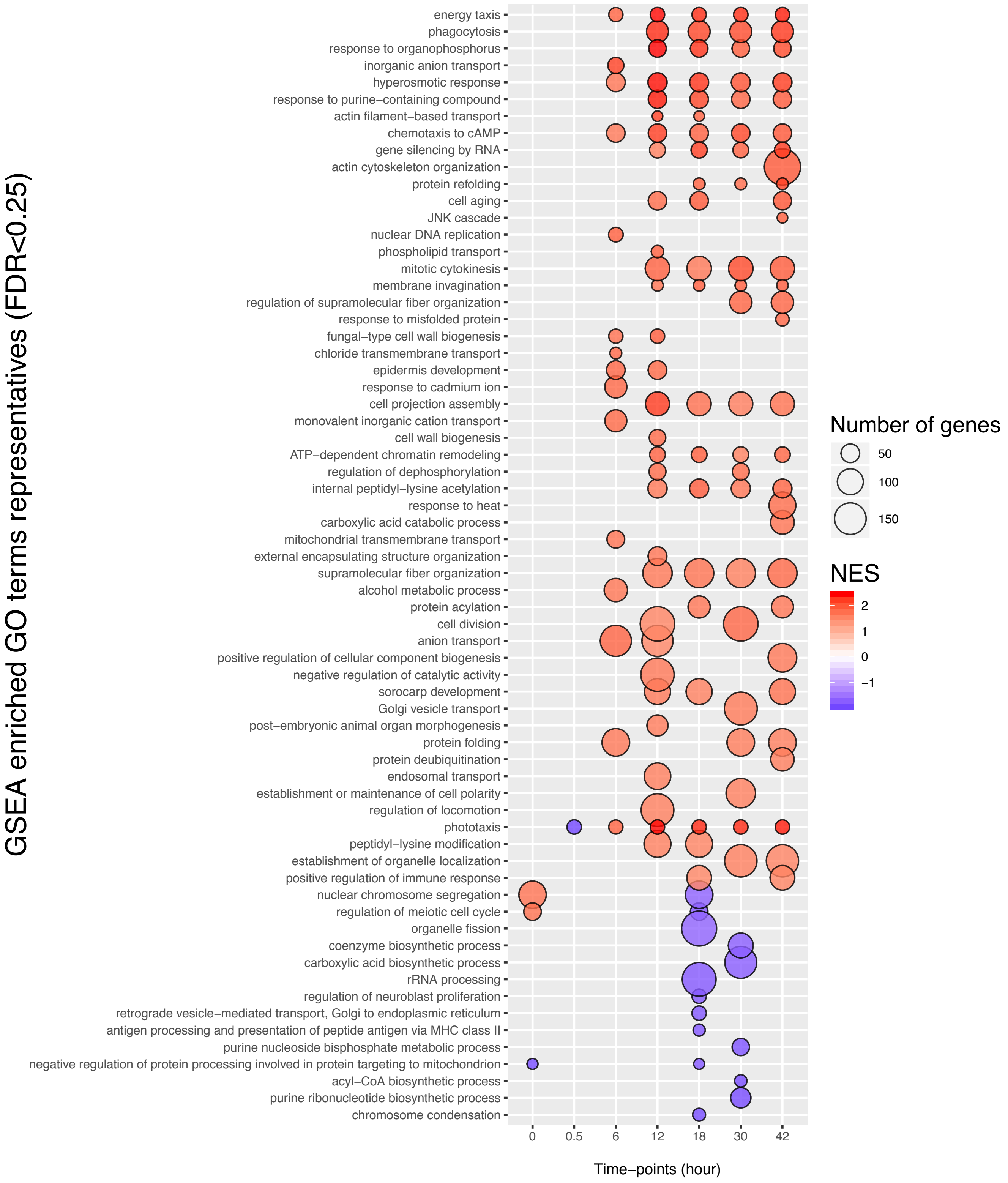

Supplementary fig. S4. Enriched GO terms for each time point using gene set enrichment analysis (GSEA). A positive normalized enrichment score (NES) indicates that a gene set is enriched in the list of genes up-regulated by competition, whereas a negative NES indicates that the gene set is enriched in the list of down-regulated genes. Size of the circle indicates number of genes in the GO term.

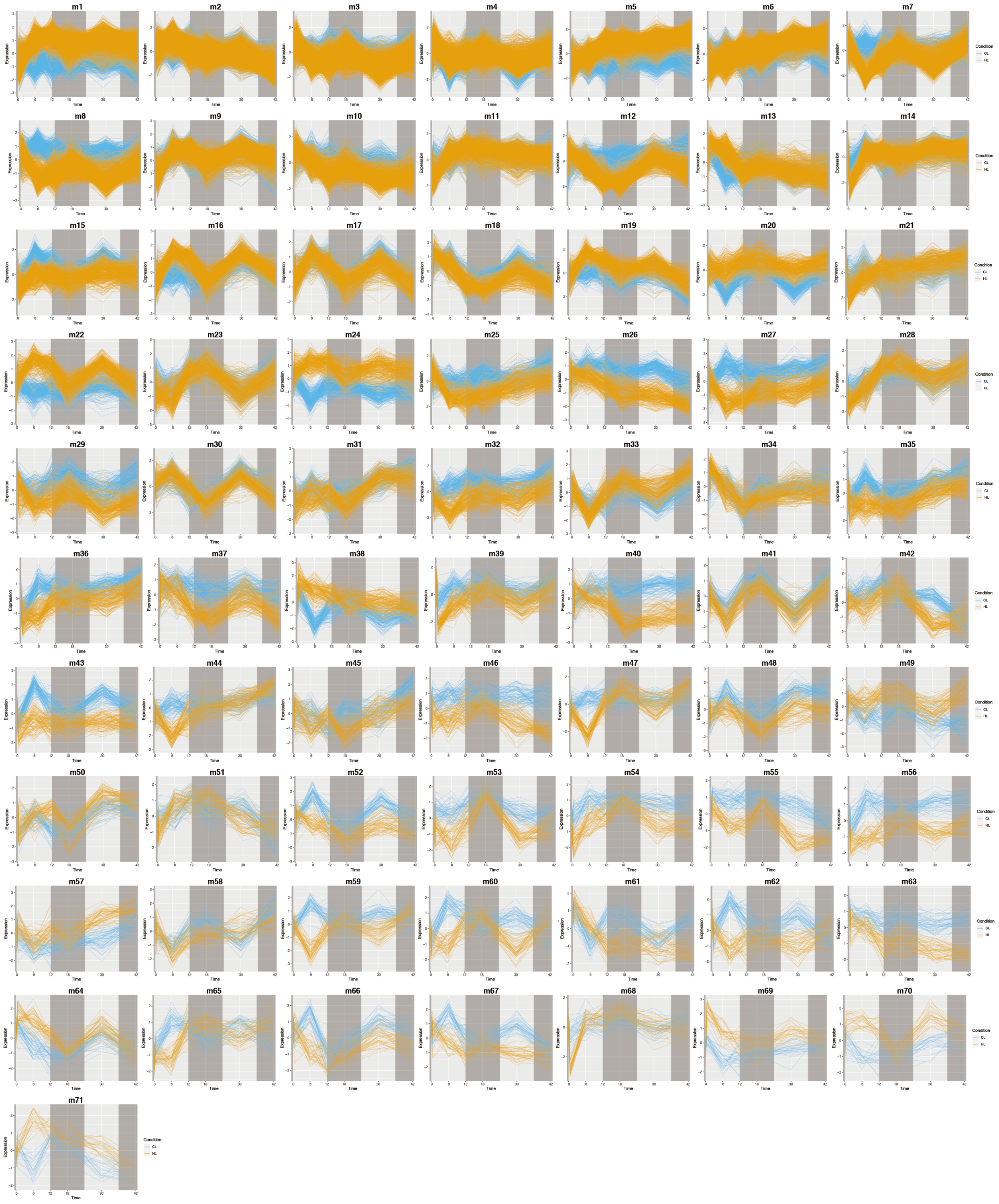

Supplementary fig. S5. Expression patterns of modules. Log expression count was z-score normalized.

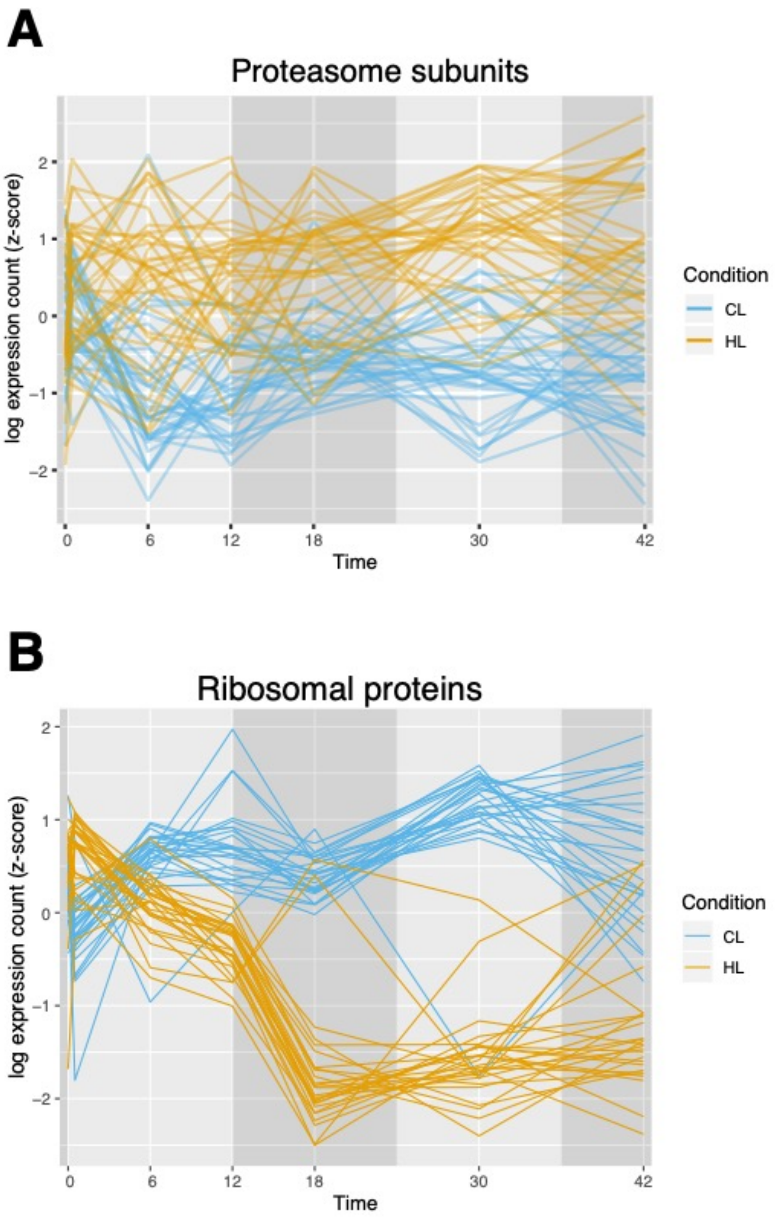

Supplementary fig. S6. (a) Expression pattern of peroxisome subunits. (b) Expression pattern of ribosomal proteins in KR01. Asterisks (*) indicate significant difference in expression count by DESeq2 (p-value < 0.05 and $|\log_2$ (fold change)$| > 1$).

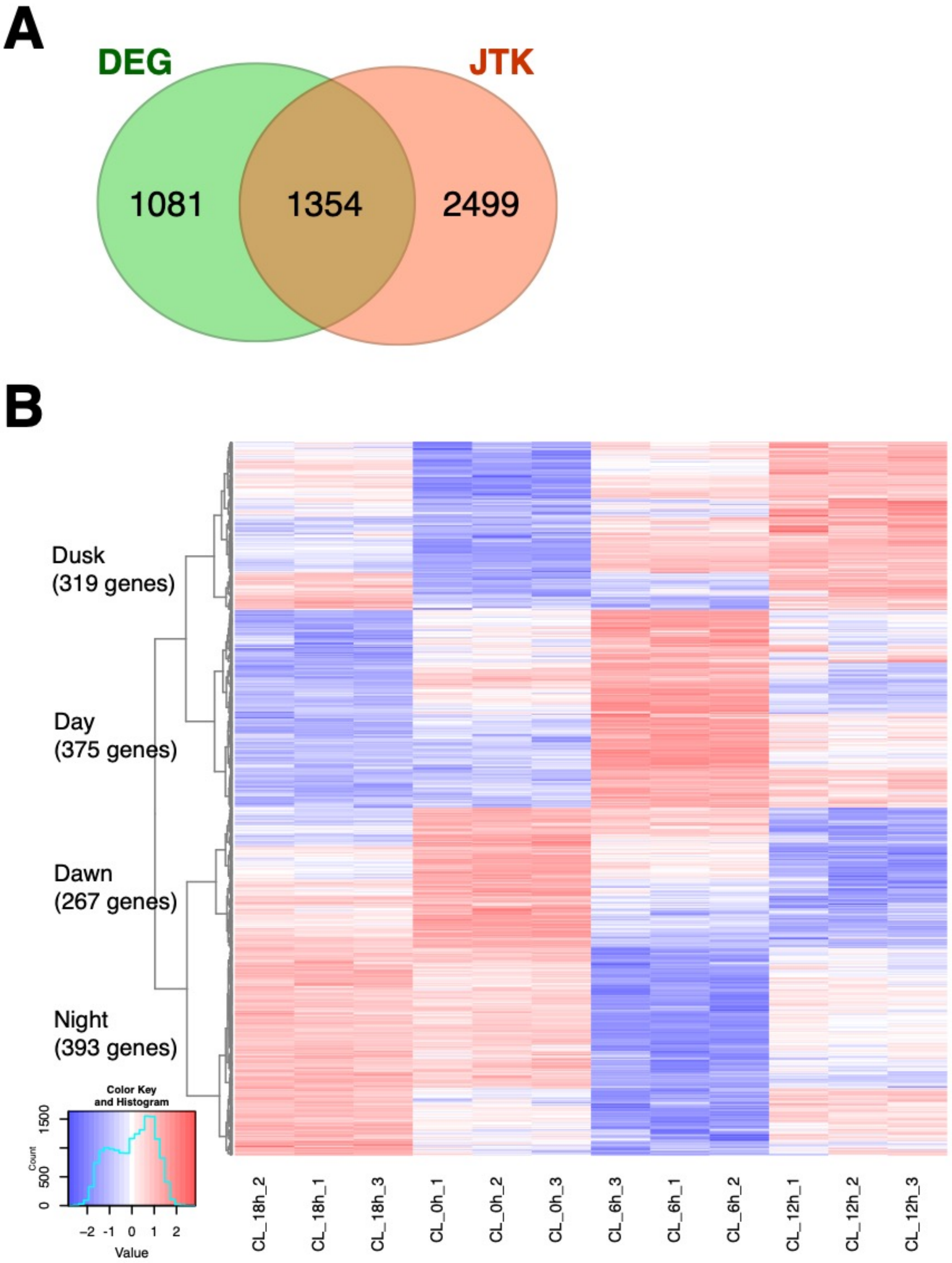

Supplementary fig. S7. (A) Venn diagram showing overlap of 2,435 DEG genes (|$\log_2$ (fold change)| > 1 between any two time points) and the 3,853 JTK rhythmic genes. (B) Expression heat map of diurnal genes (overlap of 1,354 genes) using four time points in control light condition (0h, 6h, 12h, 18h). Genes are clustered based on their Euclidean distance using the regularized log transformed count data.

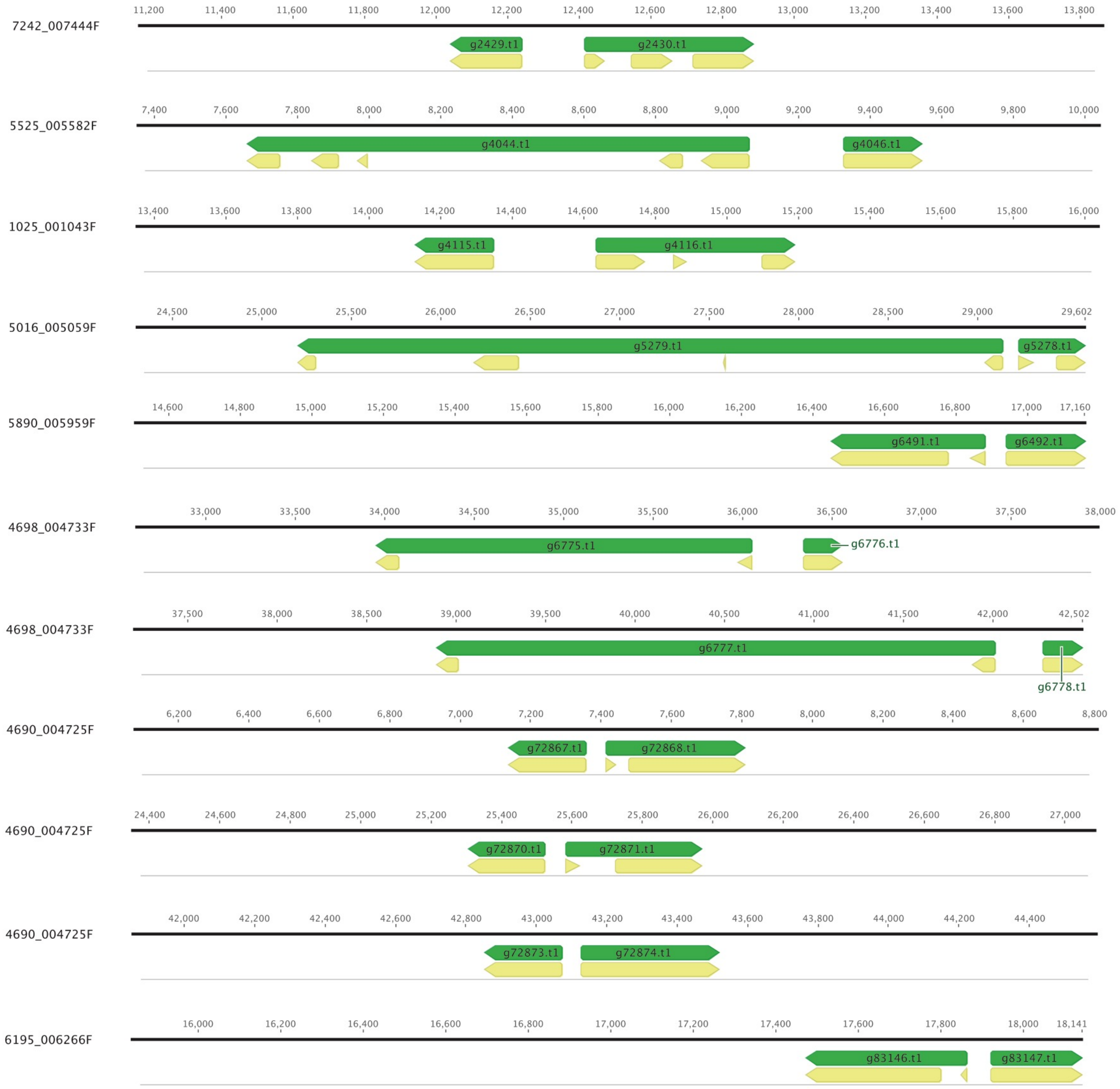

Supplementary fig. S8. Region of *hli* genes from KR01 genome showing the paired position. Green indicate gene region and yellow indicate exon structure. The position in base pairs is shown above the contig.

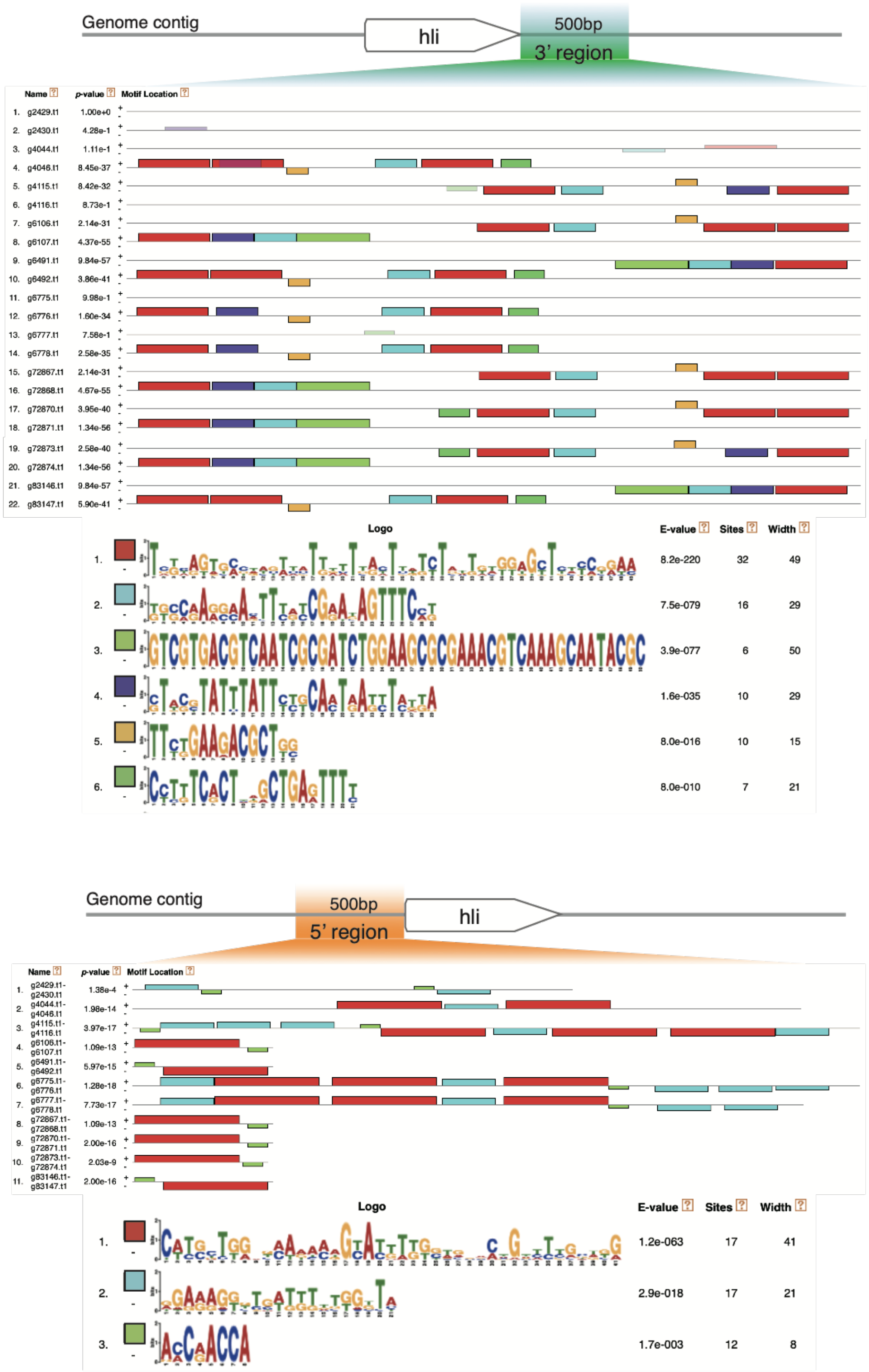

Supplementary fig. S9. Identification of motifs detected using 500 bp upstream and downstream of the *hli* genes as a training set E value (MEME) is shown. The 500 bp upstream regions are shared between paired *hli* genes. The color of small boxes in front of motif sequences match motif boxes on the 500 bp upstream and downstream region.

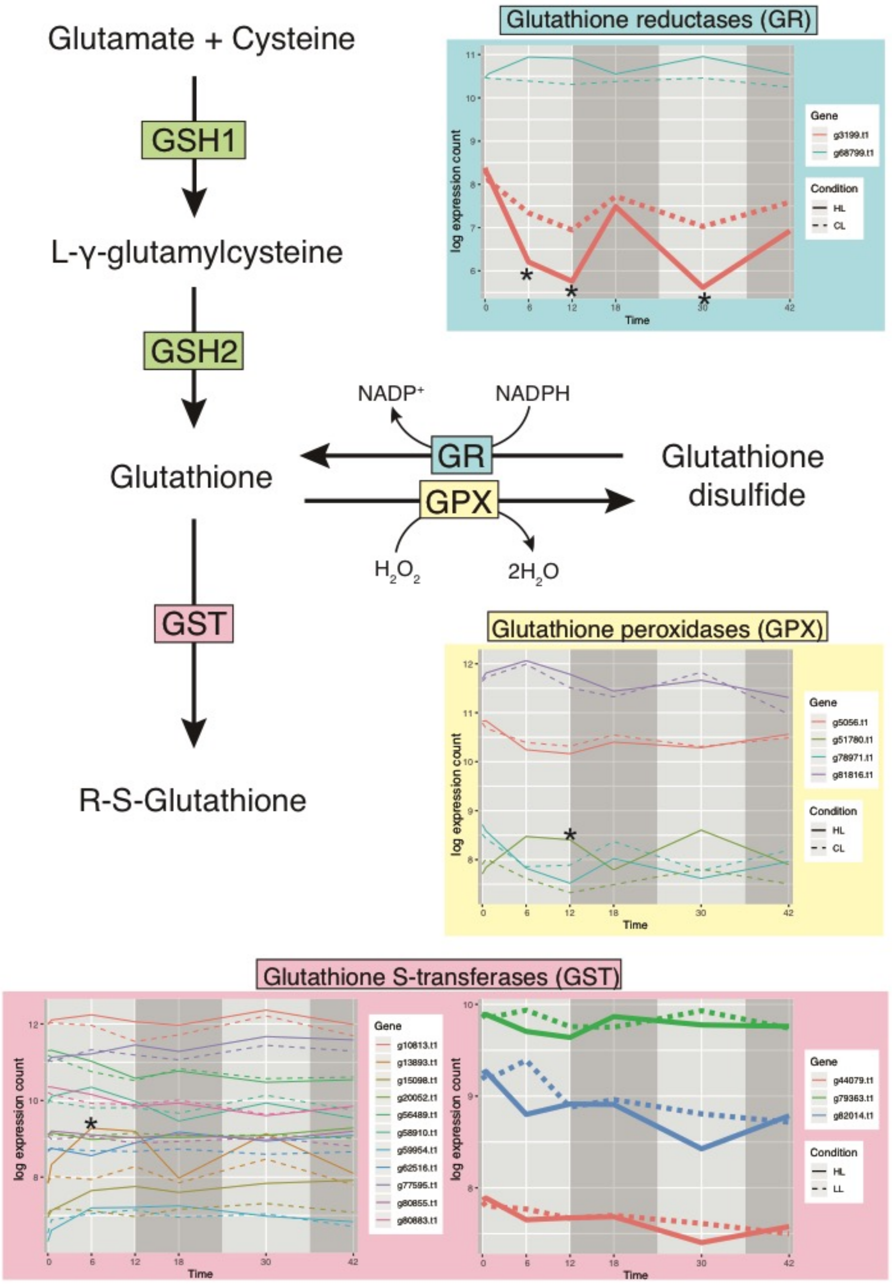

Supplementary fig. S10. Pathway diagram and gene expression of reconstructed glutathione-related processes. The graphs show expression level of each gene (color coded) with asterisks (*) indicating significant differences in expression count using DESeq2 (p-value < 0.05 and $|\log_2$ (fold change)$| > 1$). The thick lines denote proteins that contain a crTP.

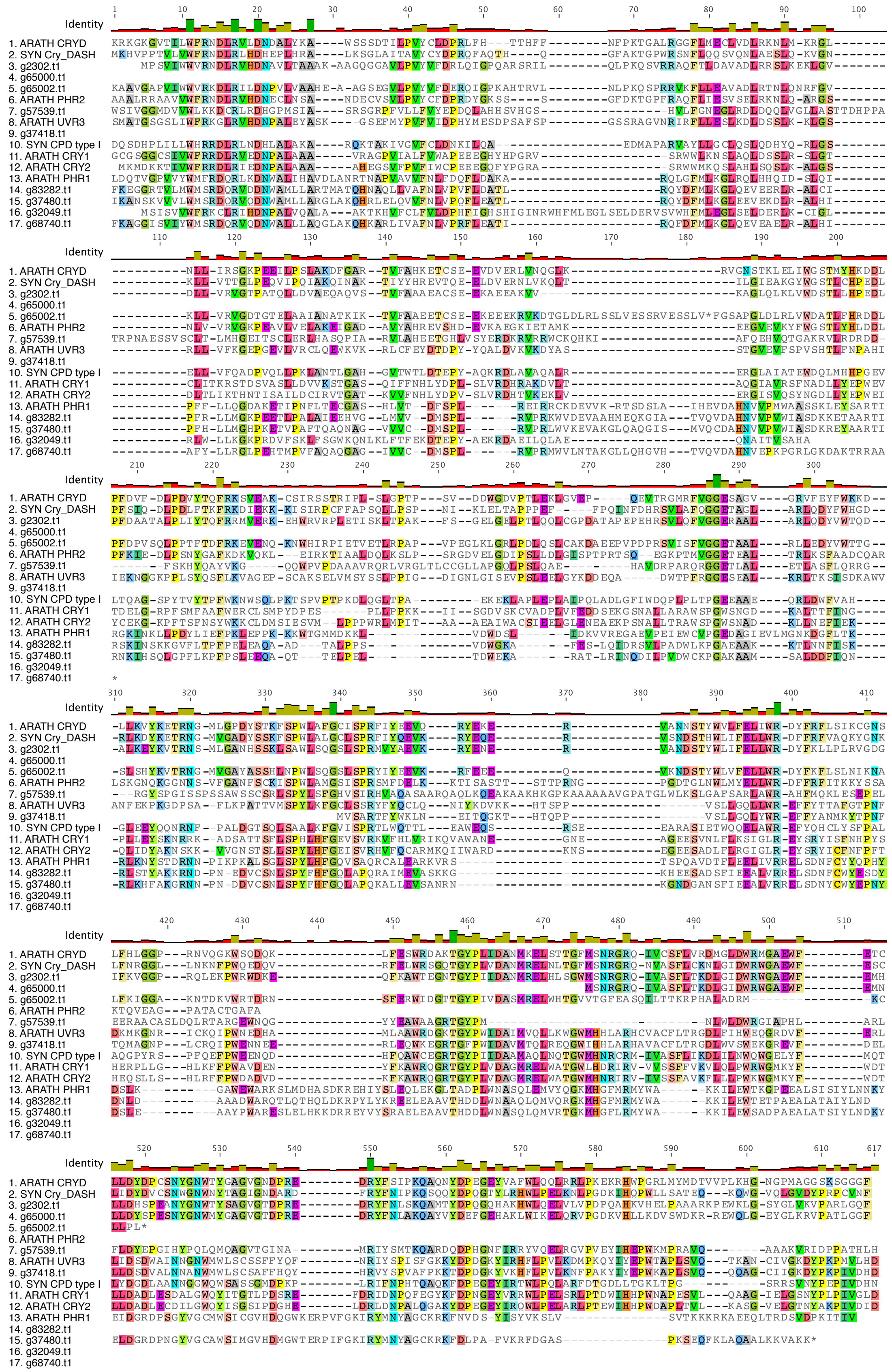
Identity
1. ARATH CRYD
2. SYN Cry_DASH
3. g2302.t1
4. g65000.t1
5. g65002.t1
6. ARATH PHR2
7. g57539.t1
8. ARATH UVR3
9. g37418.t1
10. SYN CPD type I
11. ARATH CRY1
12. ARATH CRY2
13. ARATH PHR1
14. g83282.t1
15. g37480.t1
16. g32049.t1
17. g68740.t1

Supplementary fig S11. Cryptochrome/photolyase family (CPF) in KR01. 9 CPF genes are aligned with CPF genes in *Arabidopsis thaliana* and *Synechocystis* sp. PCC 6803. Sequence information: ARATH CRYD, sp|Q84KJ5 Cryptochrome DASH chloroplastic/mitochondrial CRYD [*A. thaliana*]; SYN Cry_DASH, YP_005382952.1 DNA photolyase [*Synechocystis* sp. PCC 6803 substr.GT-I]; ARATH PHR2, sp|Q8LB72 Blue-light photoreceptor PHR2 [*A. thaliana*]; ARATH UVR3, sp|O48652 (6-4)DNA photolyase UVR3 [*A. thaliana*]; SYN CPD type I, NP_441110.1 deoxyribopyrimidine photolyase [*Synechocystis* sp. PCC 6803]; ARATH CRY1, sp|Q43125 Cryptochrome-1 CRY1 [*A. thaliana*]; ARATH CRY2, sp|Q96524 Cryptochrome-2 CRY2 [*A. thaliana*]; ARATH PHR1, sp|Q9SB00 Deoxyribodipyrimidine photo-lyase CPD typeII PHR1 [*A. thaliana*]. Conserved residues are shown in color.

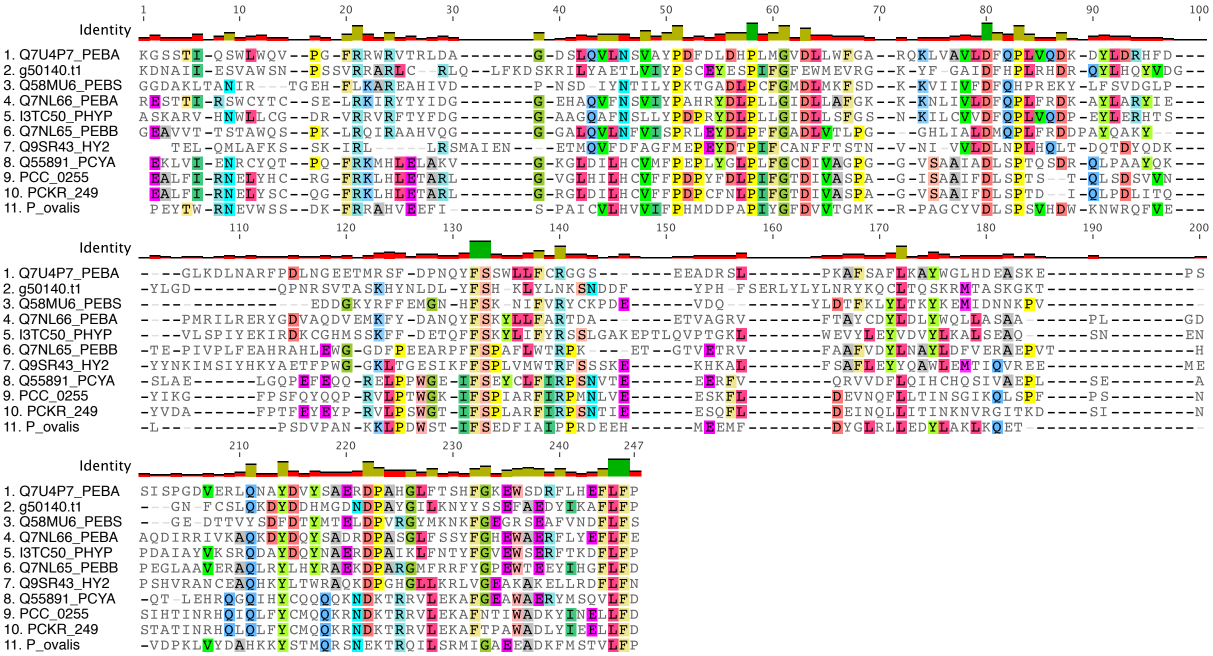

Supplementary fig S12. Alignment of ferredoxin- dependent bilin reduction (FDBR)s in *Paulinella* with other FDBR genes. Sequence information: Q7U4P7_PEBA, sp|Q7U4P7 15,16-dihydrobiliverdin:ferredoxin oxidoreductase PEBA [*Synechococcus* sp. (strain WH8102)]; Q58MU6_PEBS, sp|Q58MU6 Phycoerythrobilin synthase PEBS [*Prochlorococcus* phage P-SSM2]; Q7NL66_PEBA, sp|Q7NL66 15,16-dihydrobiliverdin:ferredoxin oxidoreductase PEBA [*Gloeobacter violaceus* (strain ATCC 29082 / PCC 7421)]; I3TC50_PHYP, tr|I3TC50 Phycourobilin synthase PHYPA [*Physcomitrella patens* subsp. patens]; Q7NL65_PEBB, sp|Q7NL65 Phycoerythrobilin:ferredoxin oxidoreductase [*Gloeobacter violaceus* (strain ATCC 29082 / PCC 7421)]; Q9SR43_HY2, sp Q9SR43 Phytochromobilin ferredoxin oxidoreductase chloroplastic HY2 [*Arabidopsis thaliana*]; Q55891_PCYA, sp|Q55891 Phycocyanobilin ferredoxin oxidoreductase PCYA [*Synechocystis* sp. (strain PCC 6803 / Kazusa)]; PCC_0255, PCC_0255 phycocyanobilin:ferredoxin oxidoreductase chromatophore encoded [*Paulinella chromatophora* CCAC 0185]; PCKR_249, PCKR_249 phycocyanobilin:ferredoxin oxidoreductase chromatophore encoded [*Paulinella micropora* KR01]; P_ovalis, SAG1_utg7180000009482.SAG1_utg7180000009482.g18.t1 [*Paulinella ovalis* sp.]. Conserved residues are shown in color.

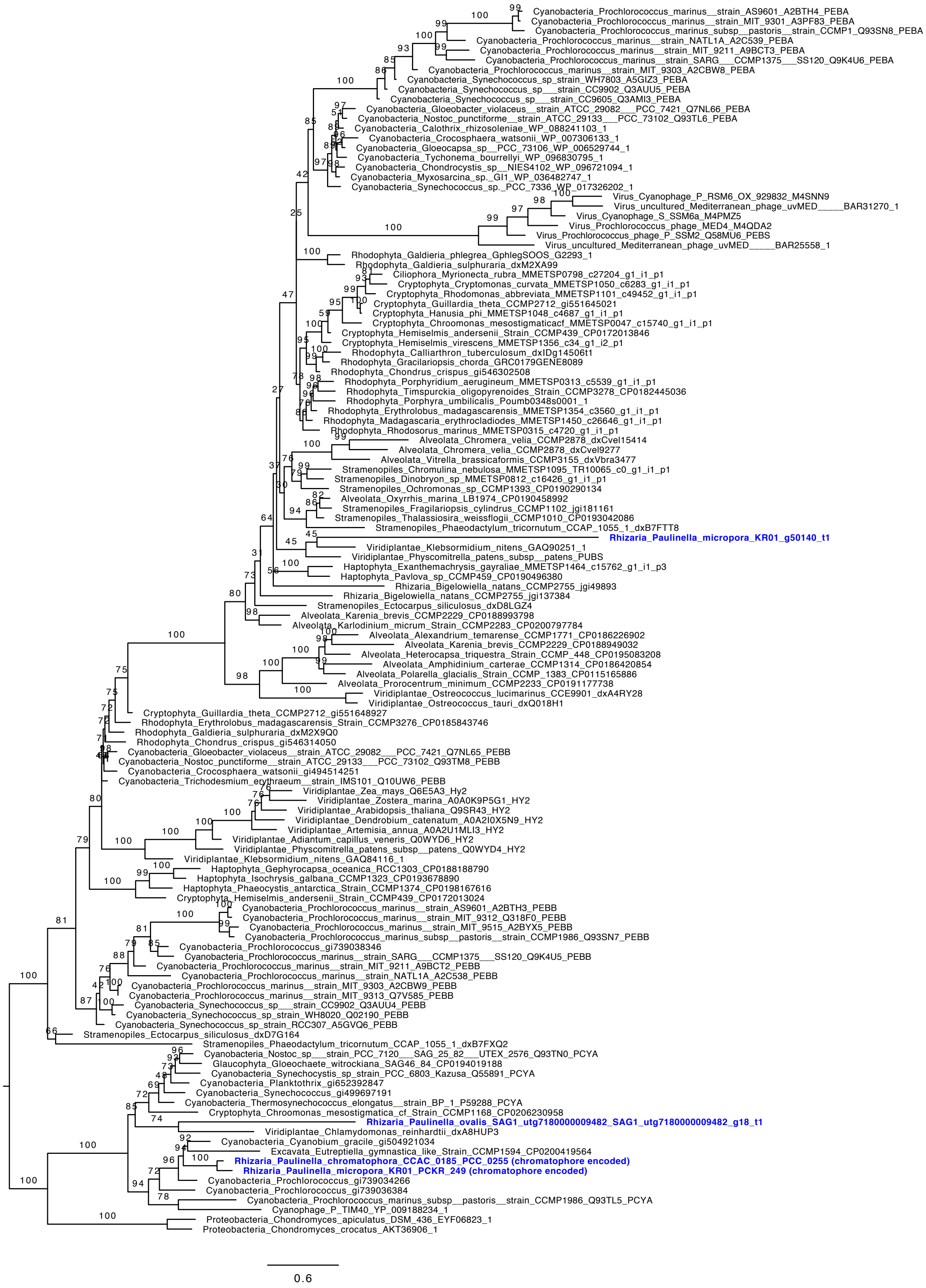
Cyanobacteria_Prochlorococcus_marinus__strain_AS9601_A2BTH4_PEBA
Cyanobacteria_Prochlorococcus_marinus__strain_MIT_9301_A3PF83_PEBA
Cyanobacteria_Prochlorococcus_marinus_subsp__pastoris__strain_CCMP1_Q93SN8_PEBA
Cyanobacteria_Prochlorococcus_marinus__strain_NATL1A_A2C539_PEBA
Cyanobacteria_Prochlorococcus_marinus__strain_MIT_9211_A9BCT3_PEBA
Cyanobacteria_Prochlorococcus_marinus__strain_SARG___CCMP1375___SS120_Q9K4U6_PEBA
Cyanobacteria_Prochlorococcus_marinus__strain_MIT_9303_A2CBW8_PEBA
Cyanobacteria_Synechococcus_sp_strain_WH7803_A5GIZ3_PEBA
Cyanobacteria_Synechococcus_sp___strain_CC9902_Q3AUU5_PEBA
Cyanobacteria_Synechococcus_sp___strain_CC9605_Q3AMI3_PEBA
Cyanobacteria_Gloeobacter_violaceus__strain_ATCC_29082___PCC_7421_Q7NL66_PEBA
Cyanobacteria_Nostoc_punctiforme__strain_ATCC_29133___PCC_73102_Q93TL6_PEBA
Cyanobacteria_Calothrix_rhizosoleniae_WP_088241103_1
Cyanobacteria_Crocosphaera_watsonii_WP_007306133_1
Cyanobacteria_Gloeocapsa_sp__PCC_73106_WP_006529744_1
Cyanobacteria_Tychonema_bourrellyi_WP_096830795_1
Cyanobacteria_Chondrocystis_sp__NIES4102_WP_096721094_1
Cyanobacteria_Myxosarcina_sp._GI1_WP_036482747_1
Cyanobacteria_Synechococcus_sp._PCC_7336_WP_017326202_1
Virus_Cyanophage_P_RSM6_OX_929832_M4SNN9
Virus_uncultured_Mediterranean_phage_uvMED_____BAR31270_1
Virus_Cyanophage_S_SSM6a_M4PMZ5
Virus_Prochlorococcus_phage_MED4_M4QDA2
Virus_Prochlorococcus_phage_P_SSM2_Q58MU6_PEBS
Virus_uncultured_Mediterranean_phage_uvMED_____BAR25558_1
Rhodophyta_Galdieria_phlegrea_GphlegSOOS_G2293_1
Rhodophyta_Galdieria_sulphuraria_dxM2XA99
Ciliophora_Myrionecta_rubra_MMETSP0798_c27204_g1_i1_p1
Cryptophyta_Cryptomonas_curvata_MMETSP1050_c6283_g1_i1_p1
Cryptophyta_Rhodomonas_abbreviata_MMETSP1101_c49452_g1_i1_p1
Cryptophyta_Guillardia_theta_CCMP2712_gi551645021
Cryptophyta_Hanusia_phi_MMETSP1048_c4687_g1_i1_p1
Cryptophyta_Chroomonas_mesostigmaticacf_MMETSP0047_c15740_g1_i1_p1
Cryptophyta_Hemiselmis_andersenii_Strain_CCMP439_CP0172013846
Cryptophyta_Hemiselmis_virescens_MMETSP1356_c34_g1_i2_p1
Rhodophyta_Calliarthron_tuberculosum_dxIDg14506t1
Rhodophyta_Gracilariopsis_chorda_GRC0179GENE8089
Rhodophyta_Chondrus_crispus_gi546302508
Rhodophyta_Porphyridium_aerugineum_MMETSP0313_c5539_g1_i1_p1
Rhodophyta_Timspurckia_oligopyrenoides_Strain_CCMP3278_CP0182445036
Rhodophyta_Porphyra_umbilicalis_Poumb0348s0001_1
Rhodophyta_Erythrolobus_madagascarensis_MMETSP1354_c3560_g1_i1_p1
Rhodophyta_Madagascaria_erythrocladiodes_MMETSP1450_c26646_g1_i1_p1
Rhodophyta_Rhodosorus_marinus_MMETSP0315_c4720_g1_i1_p1
Alveolata_Chromera_velia_CCMP2878_dxCvel15414
Alveolata_Chromera_velia_CCMP2878_dxCvel9277
Alveolata_Vitrella_brassicaformis_CCMP3155_dxVbra3477
Stramenopiles_Chromulina_nebulosa_MMETSP1095_TR10065_c0_g1_i1_p1
Stramenopiles_Dinobryon_sp_MMETSP0812_c16426_g1_i1_p1
Stramenopiles_Ochromonas_sp_CCMP1393_CP0190290134
Alveolata_Oxyrrhis_marina_LB1974_CP0190458992
Stramenopiles_Fragilariopsis_cylindrus_CCMP1102_jgi181161
Stramenopiles_Thalassiosira_weissflogii_CCMP1010_CP0193042086
Stramenopiles_Phaeodactylum_tricornutum_CCAP_1055_1_dxB7FTT8
Rhizaria_Paulinella_micropora_KR01_g50140_t1
Viridiplantae_Klebsormidium_nitens_GAQ90251_1
Viridiplantae_Physcomitrella_patens_subsp__patens_PUBS
Haptophyta_Exanthemachrysis_gayraliae_MMETSP1464_c15762_g1_i1_p3
Haptophyta_Pavlova_sp_CCMP459_CP0190496380
Rhizaria_Bigelowiella_natans_CCMP2755_jgi49893
Rhizaria_Bigelowiella_natans_CCMP2755_jgi137384
Stramenopiles_Ectocarpus_siliculosus_dxD8LGZ4
Alveolata_Karenia_brevis_CCMP2229_CP0188993798
Alveolata_Karlodinium_micrum_Strain_CCMP2283_CP0200797784
Alveolata_Alexandrium_temarense_CCMP1771_CP0186226902
Alveolata_Karenia_brevis_CCMP2229_CP0188949032
Alveolata_Heterocapsa_triquestra_Strain_CCMP_448_CP0195083208
Alveolata_Amphidinium_carterae_CCMP1314_CP0186420854
Alveolata_Polarella_glacialis_Strain_CCMP_1383_CP0115165886
Alveolata_Prorocentrum_minimum_CCMP2233_CP0191177738
Viridiplantae_Ostreococcus_lucimarinus_CCE9901_dxA4RY28
Viridiplantae_Ostreococcus_tauri_dxQ018H1
Cryptophyta_Guillardia_theta_CCMP2712_gi551648927
Rhodophyta_Erythrolobus_madagascarensis_Strain_CCMP3276_CP0185843746
Rhodophyta_Galdieria_sulphuraria_dxM2X9Q0
Rhodophyta_Chondrus_crispus_gi546314050
Cyanobacteria_Gloeobacter_violaceus__strain_ATCC_29082___PCC_7421_Q7NL65_PEBB
Cyanobacteria_Nostoc_punctiforme__strain_ATCC_29133___PCC_73102_Q93TM8_PEBB
Cyanobacteria_Crocosphaera_watsonii_gi494514251
Cyanobacteria_Trichodesmium_erythraeum__strain_IMS101_Q10UW6_PEBB
Viridiplantae_Zea_mays_Q6E5A3_Hy2
Viridiplantae_Zostera_marina_A0A0K9P5G1_HY2
Viridiplantae_Arabidopsis_thaliana_Q9SR43_HY2
Viridiplantae_Dendrobium_catenatum_A0A2I0X5N9_HY2
Viridiplantae_Artemisia_annua_A0A2U1MLI3_HY2
Viridiplantae_Adiantum_capillus_veneris_Q0WYD6_HY2
Viridiplantae_Physcomitrella_patens_subsp__patens_Q0WYD4_HY2
Viridiplantae_Klebsormidium_nitens_GAQ84116_1
Haptophyta_Gephyrocapsa_oceanica_RCC1303_CP0188188790
Haptophyta_Isochrysis_galbana_CCMP1323_CP0193678890
Haptophyta_Phaeocystis_antarctica_Strain_CCMP1374_CP0198167616
Cryptophyta_Hemiselmis_andersenii_Strain_CCMP439_CP0172013024
Cyanobacteria_Prochlorococcus_marinus__strain_AS9601_A2BTH3_PEBB
Cyanobacteria_Prochlorococcus_marinus__strain_MIT_9312_Q318F0_PEBB
Cyanobacteria_Prochlorococcus_marinus__strain_MIT_9515_A2BYX5_PEBB
Cyanobacteria_Prochlorococcus_marinus_subsp__pastoris__strain_CCMP1986_Q93SN7_PEBB
Cyanobacteria_Prochlorococcus_gi739038346
Cyanobacteria_Prochlorococcus_marinus__strain_SARG___CCMP1375___SS120_Q9K4U5_PEBB
Cyanobacteria_Prochlorococcus_marinus__strain_MIT_9211_A9BCT2_PEBB
Cyanobacteria_Prochlorococcus_marinus__strain_NATL1A_A2C538_PEBB
Cyanobacteria_Prochlorococcus_marinus__strain_MIT_9303_A2CBW9_PEBB
Cyanobacteria_Prochlorococcus_marinus__strain_MIT_9313_Q7V585_PEBB
Cyanobacteria_Synechococcus_sp___strain_CC9902_Q3AUU4_PEBB
Cyanobacteria_Synechococcus_sp_strain_WH8020_Q02190_PEBB
Cyanobacteria_Synechococcus_sp_strain_RCC307_A5GVQ6_PEBB
Stramenopiles_Ectocarpus_siliculosus_dxD7G164
Stramenopiles_Phaeodactylum_tricornutum_CCAP_1055_1_dxB7FXQ2
Cyanobacteria_Nostoc_sp___strain_PCC_7120___SAG_25_82___UTEX_2576_Q93TN0_PCYA
Glaucophyta_Gloeochaete_witrockiana_SAG46_84_CP0194019188
Cyanobacteria_Synechocystis_sp_strain_PCC_6803_Kazusa_Q55891_PCYA
Cyanobacteria_Planktothrix_gi652392847
Cyanobacteria_Synechococcus_gi499697191
Cyanobacteria_Thermosynechococcus_elongatus__strain_BP_1_P59288_PCYA
Cryptophyta_Chroomonas_mesostigmatica_cf_Strain_CCMP1168_CP0206230958
Rhizaria_Paulinella_ovalis_SAG1_utg7180000009482_SAG1_utg7180000009482_g18_t1
Viridiplantae_Chlamydomonas_reinhardtii_dxA8HUP3
Cyanobacteria_Cyanobium_gracile_gi504921034
Excavata_Eutreptiella_gymnastica_like_Strain_CCMP1594_CP0200419564
Rhizaria_Paulinella_chromatophora_CCAC_0185_PCC_0255 (chromatophore encoded)
Rhizaria_Paulinella_micropora_KR01_PCKR_249 (chromatophore encoded)
Cyanobacteria_Prochlorococcus_gi739034266
Cyanobacteria_Prochlorococcus_gi739036384
Cyanobacteria_Prochlorococcus_marinus_subsp__pastoris__strain_CCMP1986_Q93TL5_PCYA
Cyanophage_P_TIM40_YP_009188234_1
Proteobacteria_Chondromyces_apiculatus_DSM_436_EYF06823_1
Proteobacteria_Chondromyces_crocatus_AKT36906_1
0.6

Supplementary fig S13. Phylogeny of ferredoxin- dependent bilin reduction (FDBR)s in *Paulinella.* Tree built with IQTREE after automatic model selection with node support tested via 1,000 ultrafast phylogenetic bootstraps.

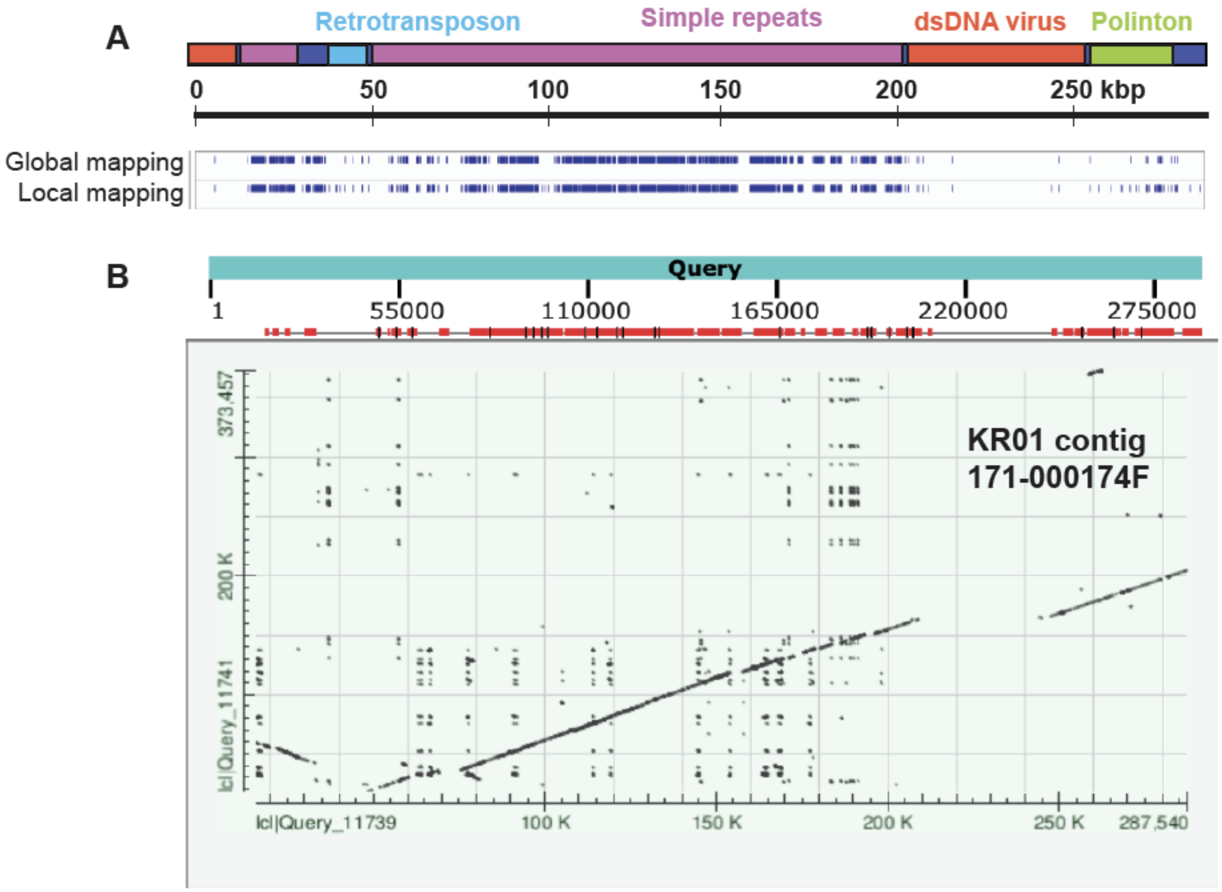

Supplementary fig. S14. (A) The dsDNA virus regions in the MYN1 genome (Scaffold1104) mapped with *P. chromatophora* reads. (B) The expanded Dot Matrix view from Blast showing the alignment of dsDNA viral regions in MYN1 (Scaffold1104) and KR01 (171-000174F).

Captions for Supplementary Tables

Supplementary table S1. Genomic sequencing information for *Paulinella micropora* KR01.

Supplementary table S2. RNA Sequencing information for *Paulinella micropora* KR01.

Supplementary table S3. Statistics of the assembled genome of *Paulinella micropora* KR01.

Supplementary table S4. Detailed information about repeat content in the *Paulinella micropora* KR01 genome.

Supplementary table S5. COG function of photosynthetic *Paulinella* gained OGFs when compared to the full set of OGFs in KR01.

Supplementary table S6. List of KR01 genes involved in signal transduction in photosynthetic *Paulinella*.

Supplementary table S7. Putative functions of EGT-derived genes.

Supplementary table S8. Functional annotation of long protein chromatophore import candidates in KR01.

Supplementary table S9. Result of differentially expressed genes (DEGs) analysis for each time-point using DESeq2 (padj < 0.05 and $|\log_2$ (fold change)$| > 1$).

Supplementary table S10. GO term and KEGG pathway enrichment (p-value < 0.05) of co-expression modules.

Supplementary table S11. Module enrichment (p-value < 0.05) of crTP/HGT/diurnal and DEG genes. Color codes: red, modules enriched with up-regulated DEGs in all time points; blue, modules enriched with up-regulated DEGs in all time points; purple, modules enriched with diurnally rhythmic genes; orange, modules enriched with crTP containing genes.

Supplementary table S12. List of high-light-inducible proteins.

Supplementary table S13. List of genes in co-expression network m22. Color codes: red, high light inducible protein; blue, glutathione metabolism genes; green, ATP-dependent zinc metalloprotease.

Supplementary table S14. List of proteosome subunit genes.

Supplementary table S15. List of ribosomal protein genes. Color codes: red, cytosolic ribosomal protein.

Supplementary table S16. List of diurnally rhythmic genes.

Supplementary table S17. GO term enrichment result (p-value < 0.05) of diurnally rhythmic genes.

Supplementary table S18. GO term and KEGG pathway enrichment (p-value < 0.05) of diurnally rhythmic genes shared with other photosynthetic species.

Supplementary table S19. List of peroxiredoxins in KR01.

Supplementary table S20. List of glutathione related genes. Color codes: green, glutathione synthetase; blue, glutathione reductase; yellow, glutathione peroxidase; pink, glutathione S-transferase.

Supplementary Table S21. List of superoxide dismutases in KR01.

Supplementary Table S22. List of cryptochrome/photolyase family genes in KR01.
