# Supplementary material for: Amoeba Genome Reveals Dominant Host Contribution to Plastid Endosymbiosis
Source: Mol Biol Evol. 2020 Aug 13;38(2):344–57. doi: 10.1093/molbev/msaa206 (PMC7826189; doi:10.1093/molbev/msaa206)
Supplement: msaa206_Supplementary_Data [file Response to reviewers_R1.pdf › msaa206-suppl_data/Response to reviewers_R1.pdf]

Editors' comments to the author:
This manuscript has been evaluated by two expert reviewers. We apologize for the delays in returning this decision. Both reviewers felt that this manuscript presented an important genome resource, with the significance of this resource both reduced and increased by the availability of a similar genome. We would be receptive to considering a highly revised version of this manuscript that responds to reviewer concerns and presents a clear biological focus for the manuscript that will be of broad interest to our readers.

Reviewers' comments:
Reviewer: 1
Comments to the Author
This work presents a genome assembly for Paulinella micropora FK01, an obscure Rhizarian amoeba that is also a very important organism indeed for studying the establishment of endosymbioses. Somewhat unusually, the analysis presented here is partially designed to refute the analysis of a newly sequenced genome of another strain of P. micropora, with the latter paper currently existing as a publicly available preprint. Overall, the availability of these genomes is a major advance for those interested in the origins of eukaryotic photosynthesis. The analysis of light-regulated gene expression is also valuable. There are a few aspects of the manuscript that would benefit from revision.

1. The authors discuss plastid genome reduction extensively, but they do not really integrate the obvious point that the Paulinella chromatophore is not only more recent but also is descended from a free-living cyanobacterium that had itself undergone extensive genome reduction BEFORE endosymbiosis. They also do not really leverage the known properties of that cyanobacterial ancestor. For example, how does knowledge of the cyanobacterial ancestral lineage inform our understanding of light regulation in Paulinella? Are there similarities to high-light/low-light Synechococcus strains?

This reviewer raises an important point about genome size that we have now addressed in the revised manuscript.
Lines 63-74:
"The α-cyanobacteria contain the diverged *Prochlorococcus* clade with some (free-living) species having undergone extensive genome reduction (e.g., 1.8 Mbp genome size, 1884 protein coding regions in *P. marinus* SS120; Dufresne et al. 2003). However, the chromatophore is resolved in phylogenetic trees as a sister to the entire *Synechococcus*/ *Prochlorococcus* clade or in a basal position within *Synechococcus* (Marin et al. 2007; Yoon et al. 2009). Calculation of the number of orthologous gene families (OGFs) in the chromatophore ancestor suggests this number is 2502 (Lhee et al. 2019). Therefore, it is important to note that although the likely chromatophore donor had a smaller genome than many cyanobacteria, it was not a nearly minimal genome as reported for the low-light adapted *P. marinus* SS120 (Dufresne et al. 2003). In comparison, the core genome of marine *Synechococcus* comprises 1572 gene families that includes a wide diversity of ecotypes (Dufresne et al. 2008)."

Regarding the second important point about light regulation, unfortunately, knowledge of light regulation pathways in the unknown α-cyanobacterial (ecotype) ancestor of the chromatophore is not highly informative about this process in *Paulinella*. This because of the significant gene losses that have occurred in the chromatophore genome and the large numbers of novel genes (described in our paper) that have become light-regulated to regulate this process. Much of our knowledge at this point suggests that beyond a basic high light response mechanism of ancestral origin (that we now discuss more fully in the paper, see below), many of the other pathways are novel or the expected genes show a different expression pattern when compared to plants and algae (also, see below). These results point to the need for genetic dissection to understand gene functions (work in progress). As

shown, the high light inducible proteins (HLIs) that are encoded in the nuclear genome of *Paulinella* were inherited from the cyanobacterial donor. It is relevant that Bhaya et al. (2002) showed high-light-adapted *Prochlorococcus* Med4 to have the smallest genome (1.7 Mbp), yet this species encodes more than twice the number of *hli* genes as moderate- or low-light adapted α-cyanobacteria. This result suggests that gene numbers alone do not explain the high light response mechanism because *Paulinella* lives in a low light environment yet encodes dozens of *hli* genes. These differences point to novelty in the amoeba that we have yet to understand.

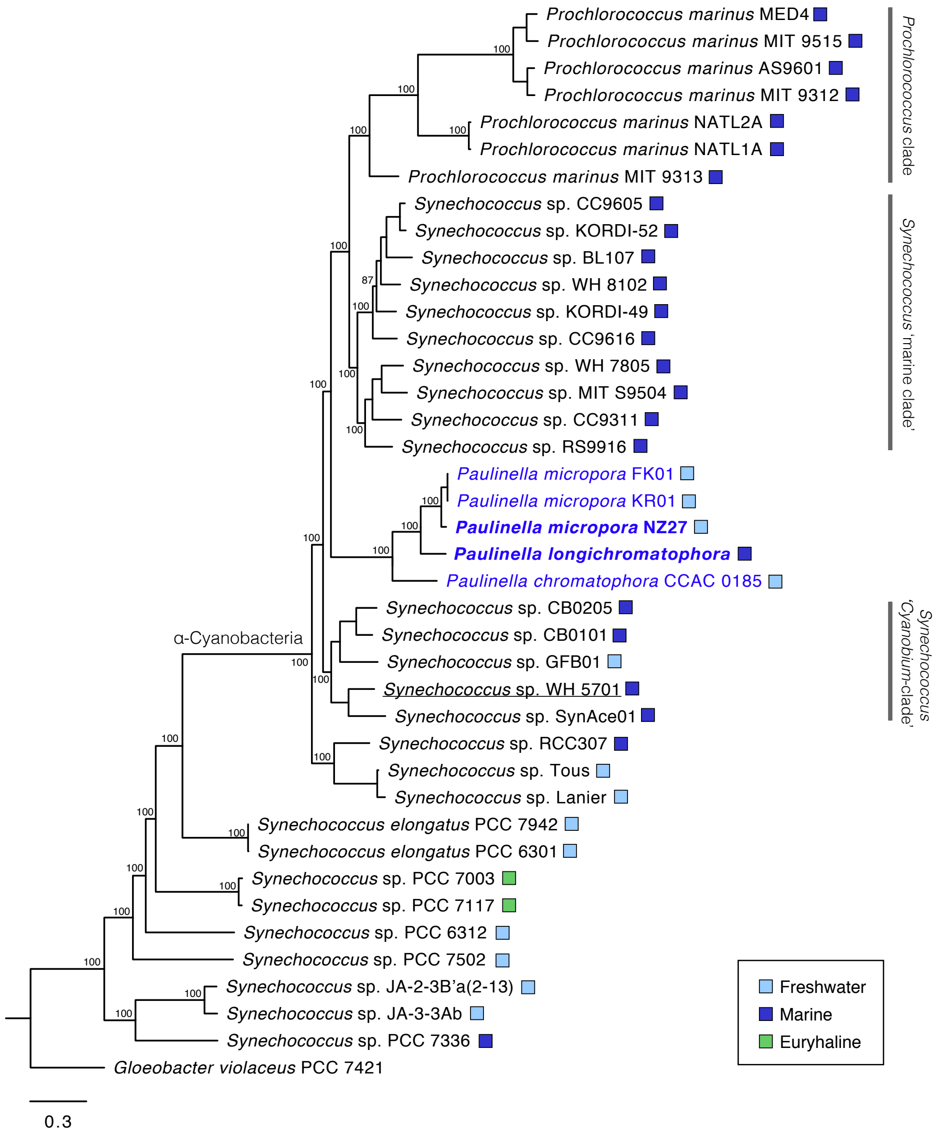

Figure S1. Phylogenomics of cyanobacteria and photosynthetic *Paulinella*. A total of 406 conserved genes were used to generate a maximum-likelihood phylogenomic tree. *Gloeobacter violaceus* PCC 7421 were used as the outgroup.

2. One example of a light-regulated gene is described in the manuscript as the ferredoxin-dependent bilin reductase (FDBR) PebA, with a vague description of the function of PebA. This discussion is problematic on multiple levels. First, the protein sequence under discussion has one fairly close relative in a standard BLAST search, itself uncharacterized, followed by a much more distant relationship to cyanobacterial PebA sequences. Based on published discussion of other FDBRs, in particular the work of Frankenberg-Dinkel and colleagues, the level of relatedness to PebA is too low to conclude that this is a PebA sequence. Moreover, PebA itself is part of a larger FDBR lineage that also includes other

enzymes (to date, PebS and PUBS). Hence, the authors would do well to describe this as a candidate PebA and include their reasoning for assigning it as such. Second, the authors would be well served by actually explaining what PebA is. This enzyme carries out the first step in the 2-step synthesis of phycoerythrobilin from biliverdin IX-alpha in cyanobacteria and some eukaryotic algae. Its presence in Paulinella is thus surprising, given the apparent lack of this chromophore in the organism and the lack of the other FDBR in this pathway (PebB). So again, the authors would do well to provide a bit of background and at least note that its presence is surprising if in fact it is a functional PebA. Lastly, the presence of a light-regulated FDBR in the nuclear genome is worth a bit more discussion in any case, given that the entire pathway for biosynthesis of phycocyanobilin is still present on the chromatophore genome in all photosynthetic Paulinella species examined to date.

We are grateful to this reviewer for pointing out that it is problematic (likely mistaken) to annotate g50140.t1 as PebA for the reasons that were provided. Therefore, we changed the annotation of g50140.t1 to an FDBR and added an alignment figure (supplementary fig. S12) and tree (supplementary fig. S13) to support this annotation. Added discussion about FDBRs was added to the manuscript.

Lines 316-330:
"Examples include the HGT-derived genes *murF* (UDP-N-acetylmuramoyl-tripeptide:D-Ala-D-Ala ligase) (Heijenoort 2001) that provides the missing step in peptidoglycan biosynthesis in the chromatophore and ferredoxin-dependent bilin reductase (FDBR) (Fig. 3C). Different FDBRs are involved in the breakdown of heme to different bilins that act as chromophores for light-harvesting (phycobiliproteins) and/or light sensing (phytochromes; Rockwell and Lagarias 2017) functions. It is interesting that bilin biosynthesis is present in all oxygenic photosynthetic lineages, regardless of the existence of phytochromes or phycobiliproteins (Rockwell, et al. 2014). We found two FDBRs in KR01. The first, *pcyA* is encoded on the chromatophore genome and is of cyanobacterial origin, whereas the second, the product of gene g50140.t1, is nuclear encoded and targeted to the chromatophore (supplementary fig. S12). Phylogenetic analysis of FDBRs demonstrates the independent derivation of these proteins in KR01 (supplementary fig. S13). The complete pathway for the biosynthesis of phycocyanobilin is still present on the chromatophore genome, however, down regulation of the chromatophore targeted FDBR under high light stress suggests that bilin biosynthesis in KR01 may be under nuclear regulation."

3. Why do the authors include a list of candidate phytochrome genes? None of these seem to encode the characteristic photosensory module one would expect, and the authors do not provide any explanation for why this was examined. It is well established that many eukaryotic algae lack these photoreceptors (rhodophytes, haptophytes, and the Rhizarian chlorarachniophytes, for example).

Again, we thank the reviewer for this important comment. In response, we have removed the content related to phytochromes.
Because Zhang, et al. (2017) showed that *hli* genes are induced during exposure to blue light and discussed cryptochrome-like proteins in *Paulinella chromatophora,* we added a section about the cryptochrome/photolyase family in KR01

Lines 290-310:
"Cryptochrome/photolyase family in photosynthetic Paulinella
The cryptochrome/photolyase family (CPF) is composed of flavoproteins with similar structures that display a variety of light-dependent functions. This family encompasses photolyases, blue-light activated enzymes that repair ultraviolet-light induced DNA damage, and cryptochromes, that regulate a variety of functions (e.g., circadian rhythm, developmental signals) in plants and animals (Fortunato, et al. 2015). Even though CPFs are present in non-photosynthetic organisms, in photoautotrophs, CPFs regulate light

acclimation and adaptation processes, suggesting that specific CPFs may have co-evolved with regulatory signals that control plastid function. A previous study (Zhang, et al. 2017) of *P. chromatophora* showed that nuclear encoded *hli* genes are induced during exposure to blue light (2 µmol photons m^{-2} s^{-1}) and these authors found two cryptochrome (CRY)-like encoding genes in the transcriptome assembly of this species. We identified nine CPFs in the genome of KR01, including partial and pseudogene copies (supplementary table S22, supplementary fig. S11). Interestingly, four of these genes show a diurnal expression pattern and two contain a crTP. Moreover, three CPF-encoding genes show differential expression under high light stress. In *A. thaliana,* CPFs play a key role in circadian clock regulation (Jarillo et al. 2001) and the synthesis of various photoprotective components (Kleine, et al. 2007). In cyanobacteria, Cry-DASH regulates photosynthetic activity and PSII repair (Vass, et al. 2013). Cry-DASH is localized in the chloroplast in *A. thaliana* (Kleine, et al. 2003). Using these studies as a guide, our finding that CPFs in KR01 are differentially regulated under high light stress and show a diurnal expression pattern, suggests these proteins are important players in light regulation of chromatophore functions in *Paulinella*."

4. On the role of HGT in establishing the endosymbiosis, do the authors see any relevance in known examples of unstable endosymbiosis, such as Hatena, Ross Sea dinoflagellate, Noctiluca, or Rapaza? It would seem possible that HGT events could occur early on, potentially priming an endosymbiotic event by supplying a gene function that renders the equivalent gene of the future plastid redundant and facilitates its loss. Hosts for unstable endosymbioses might still supply plastid-maintenance genes that were acquired by HGT.

This is an important question that we could not address using the current data. The role of pre-adaptation (e.g., HGTs) to "prime" the host cell for endosymbiosis is an intriguing area of research but the highly fragmented single cell genome assembly we currently have of the phagotrophic sister to photosynthetic *Paulinella*, *P. ovalis*, does not allow us to make robust genome-wide comparisons. The *P. ovalis* data contain significant bacterial contamination due to the wild origin of these phagotrophic cells. These bacterial genes cannot be positively identified as bona fide HGTs in the *P. ovalis* genome because of the small contig sizes. Further work on this front is planned once the shutdown eases. The closest we come to addressing this issue is discussion of the 206 ancestral crTP-containing proteins in the *Paulinella* clade. Of the total number of crTP-containing proteins, only 7 (3 shared by both species) are of HGT (bacterial) origin.

Reviewer: 2
Comments to the Author
The manuscript "Amoeba genome reveals dominant host contribution to plastid endosymbiosis" describes the analysis of the first successfully assembled genome of a photosynthetic Paulinella species, P. micropora strain KR01. The authors identify chromatophore targeted proteins and analyze their function and origins, suggesting only little impact of EGT on organelle control. Differential expression analysis under high light conditions in combination with co-expression network and enrichment analysis reveal a possible high light response plus a potential high light regulatory motif. Analysis of diurnally regulated genes resulted in only a small fraction of such genes in the analyzed strain. Most interestingly, the authors show that the viral regions found in two strains of P. micropora, are likely not involved in early gene transfer event of the genus Paulinella, as suggested by a recent preprint, as they are not present in P. chromatophora.

It is great to see another rhizarian genome published and the authors follow several interesting lines of investigation. However, I have some issues with the analysis represented in Figure 2 (which is also not described in the methods): the authors themselves mention the possibility of independent transit peptide evolution, nevertheless they present a phylogeny of

transit peptides (if I understand correctly, but hard to say without a description in the methods) in 2 different Paulinella species and draw a conclusion from it. I would like to see a discussion of why they think transit peptides evolve like the mature proteins and can thus be subjected to this method of analysis. Also, the figure is only partially labelled - only the supposedly orthologous pairs are labelled but not the rest, which does not validate the conclusion that there exists a “well-supported sister-group relationship between KR01 and P. chromatophora for most pairs”.
Otherwise, several time the authors are drawing conclusions from results that are hard to retrace for the reader. It might be just a matter of rewording, but please see examples below. Particularly the chapter “Differential gene expression analysis” is sometimes difficult to understand and could profit from a rewrite and some streamlining.

We agree with the reviewer that Fig. 2 may be too preliminary to draw substantive conclusions. Therefore, we have removed this analysis and figure from the revised manuscript. A more complete analysis will be presented elsewhere in the future.
In the transcriptome analysis part, to make the content easier to read, the paragraphs are rearranged and content was rewritten to focus on high light stress.

Specific comments:
General remark: line numbers would be very helpful

Line numbers have been added.

Abstract:
- “A total of 287 chromatophore targeted long-proteins were predicted in silico”

what are “long-proteins”? Define already here.

To explain the nature of long-proteins, substantial content would need to be added to the abstract. Therefore, we have removed “long” in the abstract (Line 37)

- “Gene co-expression analysis identified networks containing known high light stress response genes as well as a variety of putative “dark” genes of unknown function.”
are “dark” genes of unknown function only or somehow correlated with light/dark or diurnal functions? It is likely the former, but this could profit from some rewording in this context.

The latter, dark genes lack significant hits to known proteins in databases and therefore comprise de novo gene originations or are genes too highly diverged to identify their homologs with known functions. We have reworded this in the text (Lines 90-93).
Regarding the second point, much of our knowledge at this point suggests that beyond a basic high light response mechanism of ancestral origin (that we now discuss more fully in the paper, see below), many of the other genes/pathways are novel (i.e., dark). These results point to the need for genetic dissection to understand novel gene functions (work in progress).

- The last sentence from the abstract is not clearly represented by the rest of the manuscript. Where in the manuscript is the identification of “a storehouse of novel genes implicated in the transition from a hetetrotrophic lifestyle to phototrophy” described?

We have removed this sentence.

Results and Discussion:
- page 4, last paragraph/page 5, first paragraph: how many genes involved in “genetic information processing” are encoded in the organelle? i.e. how can the authors state that DNA replication/repair are primarily under host control? In general, what are the differences

between the chromatophores in this Paulinella micropora strain and P. chromatophora? Has the former been sequenced?

Reyes-Prieto et al. (2010) showed the significant conservation of gene order and only a handful of minor gene rearrangements between chromatophore genomes in *P. micropora* and *P. chromatophora*. Analysis of gene content revealed 66 differential gene losses that appeared to be outright gene deletions rather than endosymbiotic gene transfers to the host nuclear genome.
Both chromatophore genomes have retained the main functions of DNA replication, encoding DNA helicase (*dnaB*), the single- strand binding protein SSB, DNA primase (*dnaG*), gyrase (*gyrA* and *gyrB*), topoisomerase (*topA*), the replication initiation protein *dna*A, and an alpha-cyanobacterial set of DNA polymerase III subunits. However, the NAD-dependent DNA ligase (*ligA*) and DNA polymerase I (*polA*) are missing in the chromatophore genome and these functions are met by nuclear encoded *ligA* and *polA*. Therefore, host nuclear genes are necessary for chromatophore encoded metabolic pathways, including DNA replication/repair (Nowack et al. 2016).
See revised discussion in lines 131-138.

- page 5, paragraph 2: what are the 81 crTPs unique to KR01? Anything jumping out or not? Please state.

Nothing jumps out, see supplementary fig. S2b. These proteins have the same array of functions as the full set of crTP containing proteins.

- page 5, paragraph 2: concerning the 206 long import proteins shared between the two species: are they of the same evolutionary origins (i.e. alpha-cyanobacterial, other bacteria, host or unidentified)?

A total of 9/208 (latter number revised) shared proteins are of EGT origin and of alpha-cyanobacterial ancestry, whereas 3/208 are of other bacterial origin. The remaining 196 proteins are of host or unclear origin (Lines 152-157).

- page 5, last paragraph (see also above): “The crTP phylogeny (N-terminal ~260 amino acids) demonstrates a well-supported sister-group relationship between KR01 and P. chromatophora for most pairs” - the other pairs are not indicated, so this cannot be verified. Overall it is not clear what Figure 2 is supposed to show – some (presumably orthologous?) proteins cluster together, some do not. What are the other proteins that are not indicated? It is hard to gauge how many proteins are NOT “orthologs” via their crTP. Also, the overall branch length is quite long. Is this figure suggesting that the crTP evolved together with each protein separately - so the crTPs are behaving like mature protein sequences in evolution? The phylogenetic reconstruction is also not described in the methods. It is an interesting aspect but should be discussed! In general, targeting sequences seem to be specific to the plastid they are targeted to, not necessarily the host species - such as in dinoflagellates (e.g. Karenia) with haptophyte plastids that have retained two types of targeting sequences, one typical for the ancestral dinoflagellate plastid and one for the newer haptophyte plastid.

Figure and discussion were removed, see above.

- page 7, first line: so “among 21 hli genes ..., 15 were in module 1 and 22” - 15 each?, with module 22 containing 10 hli genes (leaving then 5 for module 1?). Why is module 1 not discussed further? Maybe not mention it then. This paragraph is a little confusing and should be rewritten.

Even though many *hli* genes are in module 1, this was the largest module, containing 1848 genes and therefore was difficult to analyze. Therefore, we used module 22 (298 genes) for

the detailed analysis. We have removed discussion about module 1 in the revised text. This section was rewritten as follows.

Lines 183-209:
"*Gene co-expression analysis*
We used gene co-expression analysis to investigate the RNA-Seq data. Gene co-expression networks can be used to associate genes of unknown function with biological processes, to prioritize candidate genes, or to identify transcriptional regulatory programs (van Dam et al. 2017). We found 71 distinct modules of gene co-expression (supplementary fig. S5). Examples include module 1 (m1) enriched in actin related GO terms, chemotaxis, and signaling, whereas "RNA processing" was enriched in m4, "potassium ion transport" in m30, and "DNA replication" in m2 (supplementary table S10). We calculated module enrichment in terms of proteins with the features crTP/HGT/diurnal expression and DEGs at each time point ($|\log_2$ (fold change)$| >1$) (supplementary table S11). Of particular interest was the "high-light-inducible" (*hli*) (Komenda and Sobotka 2016) gene cluster that includes components of the high light response "toolkit" in KR01. Among 34 *hli* genes with assigned modules, 13 were in m22 showing upregulation under high light (supplementary table S12). Besides the 13 HLI proteins (red), the m22 co-expression network contained four glutathione related proteins (blue), as well as a variety of "dark" proteins (gray) (Figs. 2D and 2E; supplementary table S13). Here, dark genes or proteins (Stephens et al. 2018; Cleves et al. 2020) have unknown functions (i.e., lack a MMseqs2 hit at *e*-value cut-off < $1e^{-5}$ against the nonredundant NCBI database) and are specific to photosynthetic *Paulinella*. Dark genes comprise de novo gene originations in the *Paulinella* clade (and potentially its sister species for which we have no genome data) or are genes too highly diverged to identify their homologs with known functions. Other genes in module m22 include ATP-dependent zinc metalloprotease (*ftsH*), which has a variety of functions (e.g., major thylakoid membrane protease) and is involved in the repair of the D1 protein in PSII under light induced damage (Shao et al. 2018). Nuclear encoded eukaryotic *ftsH* exhibited the same expression pattern as hli genes, suggesting that FtsH could be targeted to the chromatophore and is involved in D1 repair.

In addition, we found that components of the proteosome were generally up-regulated under high light, although expression differences were not significant using $DESeq_2$ ($|\log 2$ (fold change)$| > 1$) (supplementary fig. S6A; supplementary table S14). Co-expression network analysis showed that cytosolic ribosomal protein genes were down-regulated under high light stress, suggesting growth inhibition (supplementary fig. S6B; supplementary table S15). The results of this analysis provide the basis for selecting candidate genes for downstream analysis of the high light stress response in KR01."

- page 7: the sentence "These genes may provide novel genetic tools for improving photosynthesis in commercially important algae and plants." sound a bit thrown into there for good measure... Either expand on that statement or leave it.

We replaced the sentence.

Lines 207-209: "The results of this analysis provide the basis for selecting candidate genes for downstream analysis of the high light stress response in KR01."

- page 8: "or they have not yet evolved the machinery to deal with the redox stress resulting from overproduction of reactive oxygen (and other) species" - did you look for putative components of that machinery in your DE data? The reasoning is somehow indirect - cell division is shifted and therefore the cells cannot deal with redox stress? Please expand your reasoning.

We added a discussion of antioxidant defense and removed the cell division discussion to focus on the high light stress response in KR01.

Lines 244-288:

"Antioxidant defense in photosynthetic Paulinella

Photosynthetic species have developed several strategies to cope with oxidative stress. The major components of the antioxidant system in plants include ascorbic acid, glutathione, and tocochromanols. Genes encoding antioxidant enzymes also come into play, such as catalase (*cat*), superoxide dismutase (*sod*), and peroxiredoxins (*prx*), among others (Szymańska, et al. 2017). In cyanobacteria, genes encoding flavodiiron proteins (*flv*), an orange carotenoid protein (*ocp*), and Hlips (*hli*) are upregulated to protect against excess light energy (Muramatsu and Hihara 2012). In the case of KR01*, hli* (supplementary table S12)*, prx* (supplementary table S19)*,* and glutathione related genes (supplementary table S20) exhibit differential expression under high light stress condition. It is not clear whether ascorbic acid and tocochromanols are present in KR01 because we could not find all the genes involved in their biosynthesis. Antioxidant enzyme genes such as *cat, flv, ocp* were also not found in our dataset. However, *sod* was present, although its expression was not significantly altered under high light stress (supplementary table S21).

Expansion of the *hli* gene family and upregulation under high light stress suggests that these genes play an important role in the high light stress response in KR01*.* To identify a potential high light regulatory motif in KR01, we searched 500 bp upstream and downstream of each *hli* gene in the genome data (Materials and Methods). Many *hli* genes were in pairs and some had not previously been functionally annotated (supplementary fig. S8). Conserved motifs were discovered (supplementary fig. S9), suggesting a potential regulatory function for these sequences.

Glutathione metabolism genes appear to play an important role under high light stress because four genes were in m22 and exhibited the same expression pattern as *hli* genes and four other genes had crTPs (supplementary table S20). Glutathione is an abundant metabolite in plants that scavenges OH and singlet oxygen, may protect enzyme thiol groups, and is also involved in signal transduction (Foyer and Noctor 2005). After glutathione is biosynthesized by GSH1 (glutamate--cysteine ligase) and GSH2 (glutathione synthetase), it can participate in the detoxification of reactive electrophilic compounds by GPX (glutathione peroxidase) and GST (glutathione S-transferase) (Nutricati, et al. 2006; Rouhier 2010) (supplementary fig. S10). GR (glutathione reductase) reduces glutathione disulfide to glutathione using NADPH as an electron donor and the over-expression of GR leads to increases in antioxidant capacity (Foyer et al. 1995). When we searched for glutathione metabolism genes in the KR01 data, one was identified (GSH1) in the chromatophore genome, and other genes were nuclear encoded: i.e., one GSH2, two GR, four GPX, and 14 GSTs. We could distinguish two different expression patterns among glutathione metabolism genes. One was for genes encoding GR, GPX, and GST that shared the same expression pattern as *hli* in m22, whereby only GST and GPX showed a significant expression difference ($|\log 2$ (fold change)$| > 1$) under high light stress. The second pattern included genes that contain a crTP and were down regulated, whereby only GR showed a significant expression difference. In *A. thaliana*, GR, GST, and GPX are induced under high light stress (Kimura et al. 2003). As in the plant system (Gill et al. 2013), some GST and GR proteins were targeted to the chromatophore in KR01. This suggests that an analogous system for mitigating the negative impacts of high light stress has evolved in the *Paulinella* primary endosymbiosis, although chromatophore-destined proteins show a different expression pattern under high light stress than described in plants. Further study is needed to understand the glutathione-based antioxidant response in KR01*."*

- page 8: "There were however several chromatophore-targeted proteins significantly up-regulated under high light, one of which is NCED2 (9-cis-epoxycarotenoid dioxygenase) that is induced by stress and targeted to the chloroplast in Arabidopsis thaliana...This finding suggests that nuclear genes in KR01 may also play a role in controlling the expression of light-harvesting genes in the chromatophore." How is the upregulation of NCED2 correlated to light harvesting? Please explain your reasoning.

There was a problem with this description. The correlation to light harvesting concerned FDBR (previously PebA; see above) gene. We rewrote the paragraph and added more discussion.

Lines 331-339:
"In contrast to downregulated genes, there were several chromatophore-targeted proteins that were significantly up-regulated under high light. One is NCED (9-cis-epoxycarotenoid dioxygenase) that catalyzes the first step of abscisic-acid biosynthesis (Fig. 3C). Abscisic acid (ABA) is a stress-related signaling molecule reported in all kingdoms of life except Archaea (Hauser et al. 2011). In *A. thaliana,* NCED is the rate-limiting step in the ABA biosynthesis pathway. Moreover, NCED is induced by stress and targeted to the chloroplast (Xiong and Zhu 2003). In *Chlamydomonas reinhardtti,* ABA protects the PSII complex from photo-induced damage (Saradhi et al. 2000). These studies and our result point to the possibility that ABA is a stress response mechanism in KR01."

- page 9 and legend of Figure 4C: what do the authors understand under the term "phylostratum"? Please explain. Also, the figure legend ("Because the order of appearance of the clades is known (tree drawn on the top)") and the tree in the figure suggest that rhizarians appeared before Archaeplastida. Please provide a reference.

We added an explanation and removed the term "phylostratum" because it is unnecessary to make sense of the figure.

Lines 218-226:
"To gain insights into the origin and evolution of diurnally regulated genes in KR01 and in Archaeplastida, OGFs were sorted according to their relative age (Fig. 3A). A previous study (Ferrari, et al. 2019), demonstrated that despite large phylogenetic distances and dramatic differences in morphology and lifestyle, diurnally rhythmic genes of Archaeplastida species are conserved across lineages; i.e., many diurnally rhythmic genes were inherited from the common ancestor of the first algae (the Archaeplastida I component), likely due to selection to adapt to the diurnal light-dark cycle. When comparing KR01 and the Archaeplastida, diurnally rhythmic genes show a vastly different pattern even, though they share a large fraction of genes of eukaryotic origin."

The legend title of 4C (now 3A) was incorrect, therefore it was changed.
Lines 863-865: "(A) Percentage OGFs sorted according to their relative age (tree drawn on the top) for each species. There are two bars for each species; the left bar is based on whole gene sets and the right bar is based on only diurnally rhythmic genes."

The order of the rhizarian and Archaeplatida splits has no meaning in the tree. The phylogeny simply shows that they share a common eukaryotic ancestor. We have also added references for the tree in the methods (Line 565).

- Can you make the trees at least for EGTs available on a data repository? Have the HGT candidate trees been manually inspected?

All EGT and HGT trees were manually inspected and have been deposited in the paper website (http://cyanophora.rutgers.edu/P_micropora/).
